# Enhanced biosurveillance of high-consequence invasive pests: southern cattle fever ticks, *Rhipicephalus* (*Boophilus*) *microplus*, on livestock and wildlife

**DOI:** 10.1186/s13071-020-04366-x

**Published:** 2020-09-23

**Authors:** Hsiao-Hsuan Wang, William E. Grant, Pete D. Teel, Kimberly H. Lohmeyer, Adalberto A. Pérez de León

**Affiliations:** 1 Ecological Systems Laboratory, Department of Ecology and Conservation Biology, Texas A&M University, College Station, TX 77843 USA; 2 Department of Entomology, Texas A&M AgriLife Research, College Station, TX 77843 USA; 3 United States Department of Agriculture – Agricultural Research Service, Knipling-Bushland U.S. Livestock Insects Research Laboratory, and Veterinary Pest Genomics Center, Kerrville, TX 78028 USA

**Keywords:** Cattle Fever Tick Eradication Program, Host-parasite interaction, Individual-based model, Spatially-explicit model, Stochastic, Integrated tick management research, *Rhipicephalus microplus*

## Abstract

**Background:**

Some tick species are invasive and of high consequence to public and veterinary health. Socioeconomic development of rural parts of the USA was enabled partly through the eradication by 1943 of cattle fever ticks (CFT, *Rhipicephalus* (*Boophilus*) *annulatus* and *R*. (*B*.) *microplus*). The southern cattle fever ticks (SCFT, *R*. (*B*.) *microplus*) remain a real and present threat to the USA animal agriculture because they are established in Mexico. Livestock-wildlife interactions in the Permanent Quarantine Zone (PQZ) established by the century-old Cattle Fever Tick Eradication Programme (CFTEP) in south Texas endanger its operations.

**Methods:**

We describe a spatially-explicit, individual-based model that simulates interactions between cattle, white-tailed deer (WTD, *Odocoileus virginianus*), and nilgai (*Boselaphus tragocamelus*) to assess the risk for SCFT infestations across the pathogenic landscape in the PQZ and beyond. We also investigate the potential role of nilgai in sustaining SCFT populations by simulating various hypothetical infestation and eradication scenarios.

**Results:**

All infestation scenarios resulted in a phase transition from a relatively small proportion of the ranch infested to almost the entire ranch infested coinciding with the typical period of autumn increases in off-host tick larvae. Results of eradication scenarios suggest that elimination of all on-host ticks on cattle, WTD, or nilgai would have virtually no effect on the proportion of the ranch infested or on the proportions of different tick habitat types infested; the entire ranch would remain infested. If all on-host ticks were eliminated on cattle and WTD, WTD and nilgai, or cattle and nilgai, the proportions of the ranch infested occasionally would drop to 0.6, 0.6 and 0.2, respectively. Differences in proportions of the ranch infested from year to year were due to primarily to differences in winter weather conditions, whereas infestation differences among tick habitat types were due primarily to habitat use preferences of hosts.

**Conclusions:**

Infestations in nilgai augment SCFT refugia enabled by WTD and promote pest persistence across the landscape and cattle parasitism. Our study documented the utility of enhanced biosurveillance using simulation tools to mitigate risk and enhance operations of area-wide tick management programmes like the CFTEP through integrated tactics for SCFT suppression.
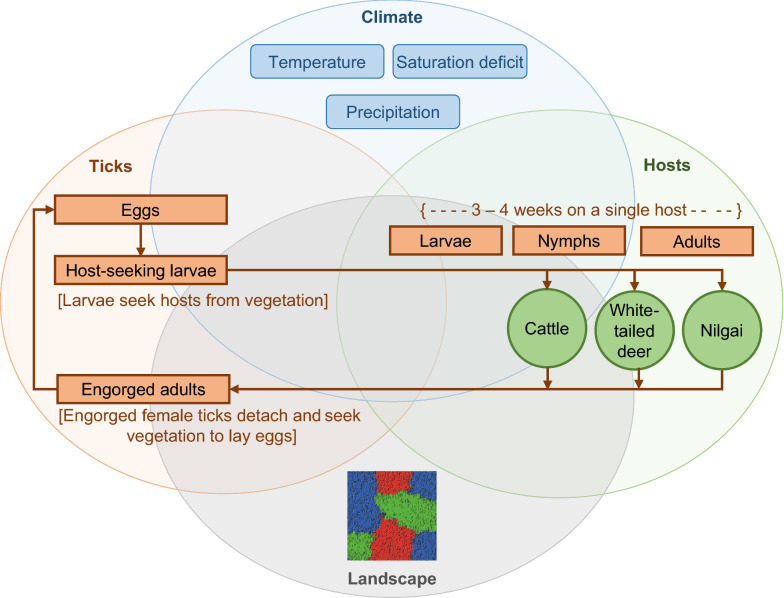

## Background

Cattle fever ticks (CFT), *Rhipicephalus* (*Boophilus*) *annulatus* and *R*. (*B*.) *microplus*, pose a significant threat to the economic security of the USA cattle industry as vectors of *Babesia bigemina* and *B. bovis*, which cause bovine babesiosis, and *Anaplasma marginale* that causes anaplasmosis [1, 2]. The Cattle Fever Tick Eradication Program (CFTEP) established in 1906 between the federal government and affected states was successful in eradicating these ticks in the USA by 1943, and thus the risk posed by bovine babesiosis [3]. However, CFT and bovine babesiosis remain endemic in neighboring Mexico and pose continuous risks to the USA livestock industry [4, 5]. To manage these risks, the CFTEP operates a permanent quarantine zone in south Texas along the border with Mexico [6] (Additional file 1: Figure S1).

Historically, success of the CFTEP depended primarily on the host specificity of CFT [7]. CFT are one-host ticks that complete their life-cycle on a single host. Upon egg-hatching, larvae have to survive environmental conditions while questing for a host. Once they latch on to a suitable host, larvae eventually attach, feed, and molt to nymphs, and then feed again to molt to adults on the same host animal. Adult females mate and engorge on the host, then detach and fall to the ground where they lay egg masses. Once considered host-specific, CFT have adapted to expand their host range in their invaded range [8]. Although historical records in the USA indicate otherwise donkeys, horses, and white-tailed deer (WTD, *Odocoileus virginianus*), were not regarded as important alternate host species until recently, thus CFT suppression efforts historically focused on domestic livestock [9, 10]. Two principal methods of tick suppression were used to eradicate CFT from the USA in the 20th century, (i) dipping all cattle, horses, donkeys, and mules systematically with a topical acaricide every 2 weeks, unless the animals and the premises were inspected and found free of CFT, and (ii) “pasture vacation”, an eradication method which involves removing all bovine and equine hosts from infested pastures and premises for a period long enough to guarantee that all off-host CFT stages died, either due to the absence of hosts or from desiccation [11].

The presence of alternate or secondary host species in the Texas-Mexico border region and beyond poses significant challenges to the CFTEP [12]. Field data indicate WTD are capable of maintaining southern cattle fever ticks (SCFT, *R*. (*B*.) *microplus*) populations in the absence of cattle, thus potentially compromising eradication efforts *via* “pasture vacation” [10, 13]. Simulation studies suggested that WTD may create SCFT refugia during acaricide treatment periods by dispersing engorged females into, and collecting host-seeking larvae from, habitat favorable for the survival and development of off-host life stages [14]. Blood from free-ranging WTD in northern Mexico tested PCR-positive for both *B. bigemina* and *B. bovis* [15], but attempts to infect WTD with a virulent *B. bovis* strain failed [16]. Currently, there are two methods for treating SCFT infesting WTD approved for use by the CFTEP: (i) treatment by feeding whole kernel corn medicated with ivermectin [17]; and (ii) use of “2-poster” bait treatment stations, which are a modification of the patented “4-poster” treatment device, which is a self-application method dispensing permethrin to the heads, necks, and ears of WTD upon contact with treated rollers during feeding on baits like whole kernel corn or pelleted protein [10].

Recent field studies have incriminated nilgai, *Boselaphus tragocamelus*, as an alternate host species for SCFT in the Texas-Mexico transboundary border region, which poses a risk for the re-emergence of CFT-borne diseases in the USA [18–20]. Nilgai, an exotic antelope species belonging to the family Bovidae and native to India, were successfully introduced to south Texas in 1941 [21]. Their similarity to cattle makes nilgai competent hosts for SCFT and potential reservoirs of the hemoparasites causing bovine babesiosis [7]. Nilgai were found to be infested with either *R. annulatus* or *R. microplus* (SCFT), as identified by the USDA, APHIS, National Veterinary Services Laboratory [10]. Blood from free-ranging nilgai in northern Mexico tested PCR-positive for both *B. bigemina* and *B. bovis*, although no SCFT were found on the nilgai from which the blood was sampled [18]. It remains unknown whether either bovine *Babesia* species can establish an infection in nilgai [19]. Further studies are needed to evaluate the biological performance of SCFT on nilgai and to determine whether nilgai can sustain a population of SCFT in the absence of cattle and/or WTD [7].

Continued success of the CFTEP requires an integrated strategy based on an interdisciplinary systems approach, which specifically includes consideration of management risks and opportunities associated with the livestock-wildlife interface [6]. Forecasting approaches, including modeling, have been advocated as a means to facilitate anticipation and enhance prevention of disease outbreaks [22–24]. Simulation models that explicitly represent key processes by which control methods intervene in the life-cycles of target species are gaining recognition as being uniquely suited for *a priori* assessments of novel eradication schemes targeted at specific aspects of a vector’s life-cycle [25]. SCFT have been the subject of a wide variety of quantitative models, which have been focused on questions dealing with both basic biology and management on four continents [26]. However, relatively few models have included explicit representation of wildlife hosts [14, 27, 28], and none have included nilgai. Addressing the knowledge gap on the dynamics of interactions between three large animal hosts on SCFT dispersal and population dynamics provides the opportunity to evaluate emerging tactics for each host species [29]. It is hypothesized that simulation modeling would inform efforts for integrated strategies to suppress SCFT populations requiring consideration of the multiplicity of wild ungulates as alternate hosts.

**Fig. 1:**
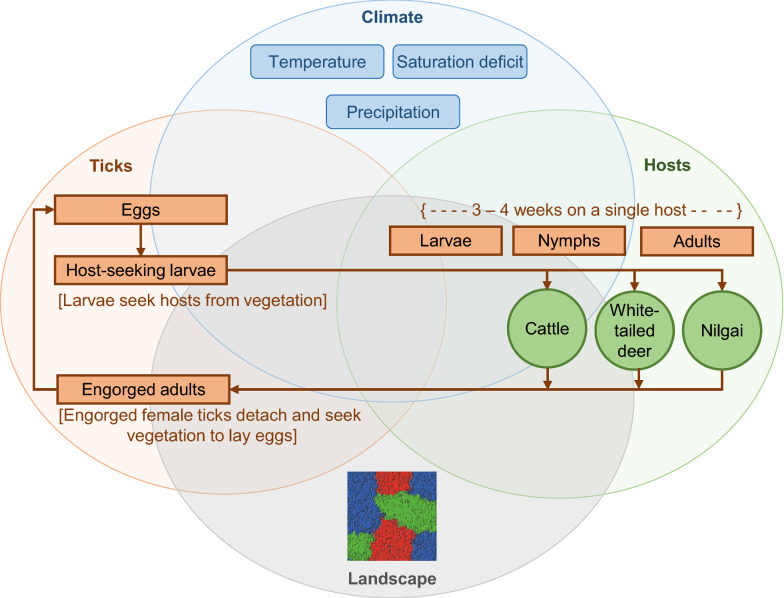
Conceptual model representing the potential role of nilgai (*Boselaphus tragocamelus*) in the maintenance of southern cattle fever tick (*Rhipicephalus* (*Boophilus*) *microplus*) populations in south Texas

In this study, the model developed by Wang et al. [14] was adapted to investigate the potential role of nilgai in sustaining SCFT populations in the USA-Mexico transboundary region comprising south Texas (Fig. 1). More specifically, we (i) extend the existing model by adding nilgai as a SCFT host, (ii) analyze sensitivity of the extended model to various alterations of a hypothetical host community including cattle, WTD, and/or nilgai, and (iii) illustrate how the extended model could be used within a management context by simulating various hypothetical infestation and eradication scenarios involving nilgai.

## Methods

### Extension of the existing model

The model of Wang et al. [14] is a spatially-explicit, individual-based, stochastic model developed to investigate the role of WTD in sustaining SCFT populations in pastures of mixed rangeland-habitat types grazed by cattle. The model is composed of 900, 1-ha landscape cells (each representing one of three different habitat types), up to several hundred individual SCFT hosts (cattle and/or WTD, depending on the scenario simulated), and local (within landscape cells) populations of SCFT (consisting of eggs, larvae, and egg-laying adults). Each host moves among landscape cells within their species-specific activity range (WTD range more widely than cattle) probabilistically based on habitat preferences and the proportions of habitat types within their individual activity ranges. A detailed description of the base model is available in Wang et al. [14]. To investigate the involvement of a third host, nilgai, we extended the model by adding code to represent nilgai as an additional host of SCFT. A list of the parameters used to represent nilgai as hosts, their baseline values, and their information sources are summarized in Additional file 1: Table S1, as is the analogous information for cattle and WTD. We re-parameterized the landscape and climate to represent a hypothetical 10,000-ha ranch under weather conditions recorded in Willacy County, Texas, USA from January 2008 through December 2018 from Climate Data Online, National Oceanic and Atmospheric Administration (https://www.ncdc.noaa.gov/cdo-web/) (Additional file 1: Figure S2). Willacy County, Texas is the focal point for SCFT infestations involving these three host species. For this project, we utilized a simulated representative landscape that was 31%, 28% and 41% of good (mesquite dominated community), fair (mixed-brush community of dominated by thorn shrubs), and poor (uncanopied forage community) habitat, respectively, with regard to the survival rates of off-host tick larvae [14, 30].

### Experimental design for sensitivity analysis scenarios

The experimental design for our sensitivity analysis consisted of three sets of scenarios in which we altered (i) the combination of host species present, (ii) the relative habitat preferences of the host species, and (iii) the relative densities of the host species. For each scenario, we ran 10 replicate stochastic (Monte Carlo) simulations. During each simulation, we monitored mean densities of off-host (potentially host-seeking) tick larvae in each of the habitats used by hosts, as well as mean numbers of adult ticks on each host species present.

Scenarios involving different combinations of host species included (i) cattle, WTD, and nilgai (baseline scenario), (ii) only cattle, (iii) only WTD, and (iv) only nilgai. Scenarios involving different relative habitat use preferences of hosts included (i) baseline preferences of all host species (Additional file 1: Table S1), as well as scenarios in which all host species used either (ii) good, (iii) fair, or (iv) poor tick habitat. Scenarios involving different densities of hosts included (i) baseline densities of all host species (Additional file 1: Table S1), as well as scenarios in which baseline densities of all host species were reduced simultaneously to (ii) 1/2, (iii) 1/4, (iv) 1/8, (v) 1/16, (vi) 1/32, (vii) 1/64, (viii) 1/128, and (ix) 1/256 of their baseline levels. We also ran scenarios in which the baseline density of each host species was reduced sequentially as just described, assuming only that single species was present.

### Hypothetical infestation and eradication scenarios

The experimental design for evaluating our proof of concept consisted of two approaches. The first was to evaluate three scenarios for initiating a tick infestation of the landscape and assessing the spatial and temporal rates of spread. The second approach was to evaluate three scenarios of SCFT eradication in which acaricide-treated hosts removed ticks from the system. For each scenario, we again ran 10 replicate stochastic (Monte Carlo) simulations. During each simulation, we monitored the number and spatial distribution of 1-ha landscape patches infested with off-host tick larvae. For the infestation scenarios, we infested the hypothetical 10,000-ha ranch by introducing one infested animal of (i) cattle, (ii) WTD, or (iii) nilgai during week 25 of 2009. We assumed the ranch was SCFT-free at the time of this infestation, and contained baseline densities of each of the three host species (Additional file 1: Table S1). Each of the three sets of scenarios involved introducing the infested host with its activity area (Additional file 1: Table S1) centered in a large patch of (i) good, (ii) fair, or (iii) poor habitat for off-host tick larvae, and also (iv) centered at the intersection of these three habitat types. For the eradication scenarios, we simulated the impact of a single acaricide treatment capable of complete and continuous elimination of all SCFT that attach to (i) cattle, (ii) WTD, (iii) nilgai, (iv) cattle and WTD, (v) WTD and nilgai, and (vi) cattle and nilgai. That is, all three host species continued to collect host-seeking larvae from the landscape, but all on-host larvae were eliminated immediately from the host species to which the acaricide was applied. The single acaricide application was applied during week 25 of 2009. Thus, in week 25 the role of individuals of the treated host species was changed from tick dispersal to tick removal.

## Results

### General trends across sensitivity analysis scenarios

General trends in mean densities of off-host tick larvae and mean numbers of adult ticks on hosts resulting from different (i) combinations of host species (Additional file 1: Figure S3), (ii) relative habitat preferences of host species (Additional file 1: Figure S4), and (iii) relative densities of the host species (Additional file 1: Figure S5) all might be summarized in the following manner. Seasonal fluctuations in mean densities of off-host tick larvae followed a bimodal pattern, with a spring increase and summer decline, followed by an autumn increase and winter decline. Increasing densities were correlated with periods of precipitation, low saturation deficits, and moderate temperatures favorable for larval survival during spring and autumn. Decreasing densities were correlated with unfavorable periods of high temperatures and saturation deficits during summer, and with unfavorable periods of low temperatures during winter, which sometimes are worsened by low precipitation and relatively higher saturation deficits. Mean numbers of adult ticks on all host species remained relatively high and constant during spring, summer and autumn (e.g. ≈ 50, ≈ 4.5 and ≈ 25 for cattle, WTD, and nilgai, respectively, during the baseline scenario), but declined to relatively low and more variable levels during winter (e.g. < 20, < 2 and < 10 for cattle, WTD, and nilgai, respectively, during the baseline scenario). Winter declines in on-host ticks were correlated primarily with off-host tick responses to low environmental temperatures, which retarded oviposition, and prolonged egg incubation, larval emergence, and host-seeking activity. The specific amplitudes and timing of seasonal fluctuations in abundances of both on- and off-host ticks varied from year-to-year in response to specific combinations of these weather parameters, and also were affected by specific characteristics of the community of host species present, which we point out below.

### Sensitivity to altering combinations of host species

Alteration of the combinations of host species present affected off-host tick larval densities noticeably, but effects on numbers of adult ticks per host were negligible. Mean larval densities were highest with all three host species present, slightly lower when only cattle were present, and much lower when only nilgai or only WTD were present (Additional file 1: Figures S3a and S5). These relative differences did not vary among the different tick habitat types (Additional file 1: Figure S6). Mean numbers of adult ticks per host were the same as those described in the previous paragraph, regardless of alterations of the host species present (Additional file 1: Figure S7b, c and d).

### Sensitivity to altering relative habitat preferences of host species

Alteration of the relative habitat preferences of host species also affected off-host tick larval densities noticeably, but, again, effects on numbers of adult ticks per host were negligible. Mean larval densities were highest when all three host species used the good tick habitat exclusively, with densities decreasing slightly with exclusive use of fair tick habitat, and more noticeably with exclusive use of poor tick habitat (Additional file 1: Figure S4). Mean numbers of adult ticks per host were the same as those described above, regardless of alterations of the relative habitat preferences of host species (Additional file 1: Figure S4b, c and d).

### Sensitivity to altering relative densities of host species

Alteration of the relative densities of all host species simultaneously affected both off-host tick larval densities and numbers of adult ticks per host noticeably. Seasonality of fluctuations and relative variations from year-to-year were not affected. However, overall, mean larval density levels decreased proportionally with decreases in host densities (Additional file 1: Figure S8a), regardless of tick habitat type (Additional file 1: Figure S7). Mean levels of numbers of adult ticks per host decreased at generally increasing rates with decreases in host densities beyond host-specific threshold levels (Additional file 1: Figure S8b, c and d). Alteration of the relative host densities when only a single host species was present affected both off-host tick larval densities and numbers of adult ticks per host in a similar manner as when all host species were present. Once again, seasonality of fluctuations and relative variations from year-to-year were not affected. However, mean larval density levels decreased proportionally with decreases in densities of the single host present (Additional file 1: Figure S9), regardless of habitat type. Mean levels of numbers of adult ticks per host also decreased at generally increasing rates with decreases in densities of the single host present, beyond host-specific threshold levels (Additional file 1: Figure S10). Minimum densities of cattle, WTD, and nilgai capable of sustaining tick populations in the absence of the other two host species were 1, 26 and 8 individuals per hectare, respectively.

### Infestation scenarios

**Fig. 2:**
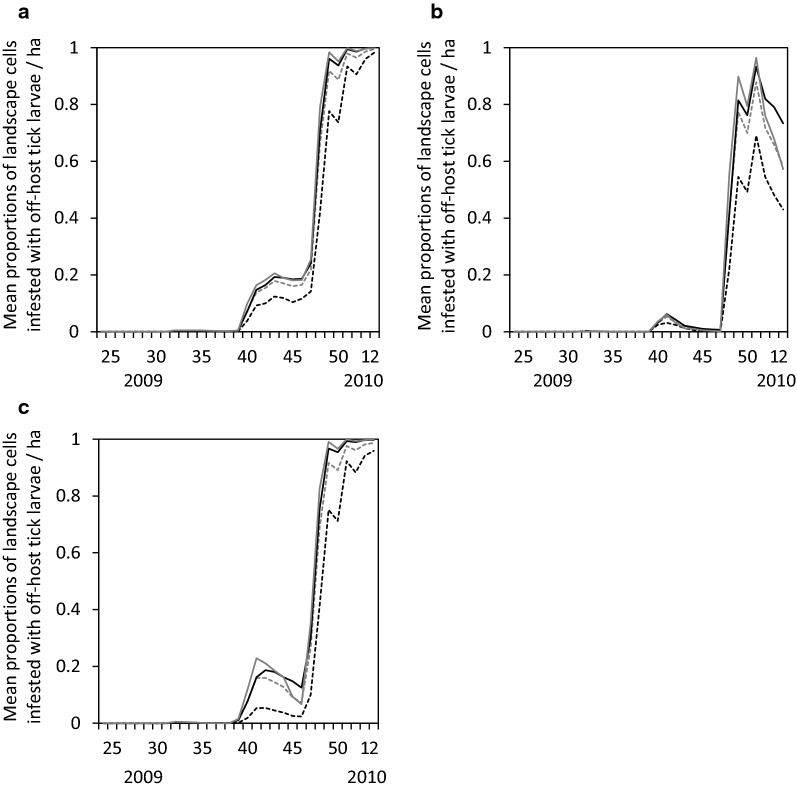
Assessment of initiation of infestation. Simulated mean proportions of landscape cells infested with off-host (potentially host-seeking) tick larvae per hectare on a hypothetical 10,000-ha ranch under weather conditions recorded in Willacy County, Texas, USA, from January 2009 through December 2018 (only week 24, 2009 through week 2, 2010 shown here): (i) on whole ranch (grey dash line), and in (ii) good (black line), (iii) fair (black dash line), and (iv) poor (grey line) tick habitats. Simulations assumed one infested head of cattle (**a**), white-tailed deer (**b**) and nilgai (**c**) was introduced at the intersection of patches of good, fair, and poor tick habitat in an otherwise SCFT-free ranch during week 25 of 2009. Tick hosts present on the ranch included cattle, white-tailed deer and nilgai. Thirty-one percent, 28 percent and 41 percent of the ranch was considered good, fair and poor habitat, respectively, for off-host tick larvae. Relative habitat use preferences of hosts for good, fair and poor tick habitats, respectively, were 0.30, 0.10 and 0.60 for cattle, 0.20, 0.40 and 0.40 for white-tailed deer, and 0.30, 0.10 and 0.60 for nilgai

The introduction of one infested head of cattle or nilgai into an otherwise tick-free landscape during mid-summer (week 25) resulted in the infestation spreading to virtually all landscape patches by late autumn (week 50) (Fig. 2a, c). Infestations introduced by one infested WTD spread more slowly (Fig. 2b) but reached virtually all landscape patches by mid**-**spring (week 8) of the following year. The slower rate of spread was due primarily to the relatively lower number of ticks carried by WTD, as well as the relatively lower fecundity of engorged female ticks that feed on WTD [14], and, hence, the lower initial size of the off-host tick population. The rate of spread was slowed further in the scenario in which the activity area of the initially-infested deer was centered in a large patch of fair tick habitat (Additional file 1: Figure S11), which was a habitat type used less frequently by the other two host species. All scenarios resulted in a phase transition from relatively fewer to relatively many infested landscape patches between weeks 47 and 48, coinciding with the typical period of autumn increases in off-host tick larvae (Additional file 1: Figures S11, S12 and S13). In all scenarios, the proportion of infested landscape patches was highest among cells classified as poor tick habitat (uncanopied forage areas) and lowest among cells classified as fair tick habitat (mixed-brush communities of thorn shrub). This counter-intuitive result reflects the simulated habitat use preferences of cattle and nilgai, which in this case spend relatively more time in poor, and relatively less time in fair, tick habitat. A time series of maps illustrating spatial spread of the infestation within the ranch can be found in Additional file 1: Figure S14.

### Eradication scenarios

**Fig. 3:**
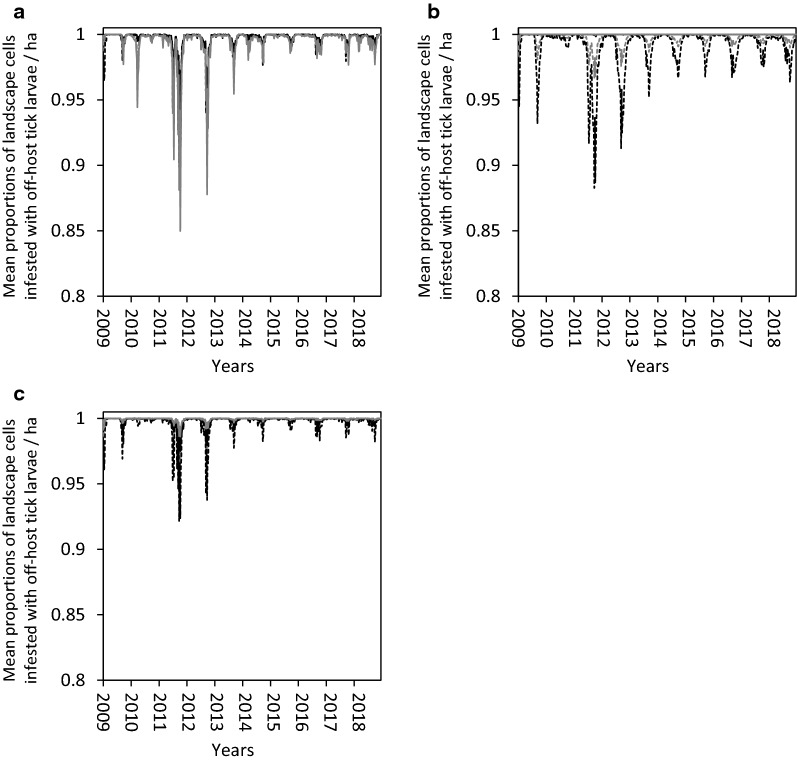
Assessment of treated hosts by single species. Simulated mean proportions of landscape cells infested with off-host (potentially host-seeking) tick larvae per hectare on a hypothetical 10,000-ha ranch under weather conditions recorded in Willacy County, Texas, USA from January 2009 through December 2018: (i) on whole ranch (grey dash line), and in (ii) good (black line), (iii) fair (black dash line), and (iv) poor (grey line) tick habitats. Simulations assumed complete and continuous elimination of all on-host ticks on cattle (**a**), white-tailed deer (**b**) and nilgai (**c**) beginning week 25 of 2009. Tick hosts present on the ranch included cattle, white-tailed deer and nilgai. Thirty-one percent, 28 percent and 41 percent of the ranch was considered good, fair and poor habitat, respectively, for off-host tick larvae. Relative habitat use preferences of hosts for good, fair and poor tick habitats, respectively, were 0.30, 0.10 and 0.60 for cattle, 0.20, 0.40 and 0.40 for white-tailed deer, and 0.30, 0.10 and 0.60 for nilgai

**Fig. 4:**
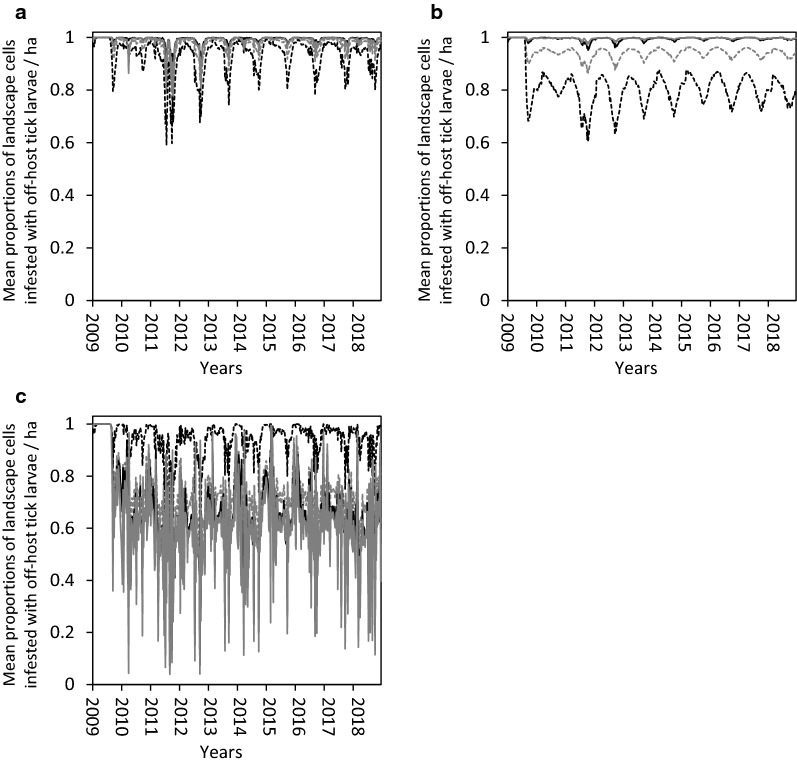
Assessment of treated hosts by paired species. Simulated mean proportions of landscape cells infested with off-host (potentially host-seeking) tick larvae per hectare on a hypothetical 10,000-ha ranch under weather conditions recorded in Willacy County, Texas, USA from January 2009 through December 2018: (i) on whole ranch (grey dash line), and in (ii) good (black line), (iii) fair (black dash line), and (iv) poor (grey line) tick habitats. Simulations assumed complete and continuous elimination of all on-host ticks on cattle and white-tailed deer (**a**), white-tailed deer and nilgai (**b**) and cattle and nilgai (**c**) beginning week 25 of 2009. Tick hosts present on the ranch included cattle, white-tailed deer and nilgai. Thirty-one percent, 28 percent and 41 percent of the ranch was considered good, fair and poor habitat, respectively, for off-host tick larvae. Relative habitat use preferences of hosts for good, fair and poor tick habitats, respectively, were 0.30, 0.10 and 0.60 for cattle, 0.20, 0.40 and 0.40 for white-tailed deer, and 0.30, 0.10 and 0.60 for nilgai

**Fig. 5:**
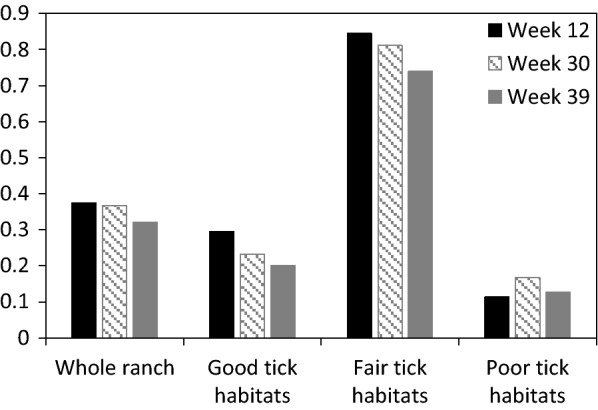
Assessment of habitat infestations when cattle and nilgai are treated for tick elimination. Simulated mean proportions of landscape cells infested with off-host (potentially host-seeking) tick larvae per hectare on a hypothetical 10,000-ha ranch during the indicated weeks of 2014: (i) on whole ranch, and in (ii) good, (iii) fair, and (iv) poor tick habitats. Simulations were run under weather conditions recorded in Willacy County, Texas, USA, from January 2009 through December 2018, and assumed complete and continuous elimination of all on-host ticks on cattle and nilgai beginning week 25 of 2009. Tick hosts present on the ranch included cattle, white-tailed deer and nilgai. Thirty-one percent, 28 percent and 41 percent of the ranch was considered good, fair and poor habitat, respectively, for off-host tick larvae. Relative habitat use preferences of hosts for good, fair and poor tick habitats, respectively, were 0.30, 0.10 and 0.60 for cattle, 0.20, 0.40 and 0.40 for white-tailed deer, and 0.30, 0.10 and 0.60 for nilgai

**Fig. 6:**
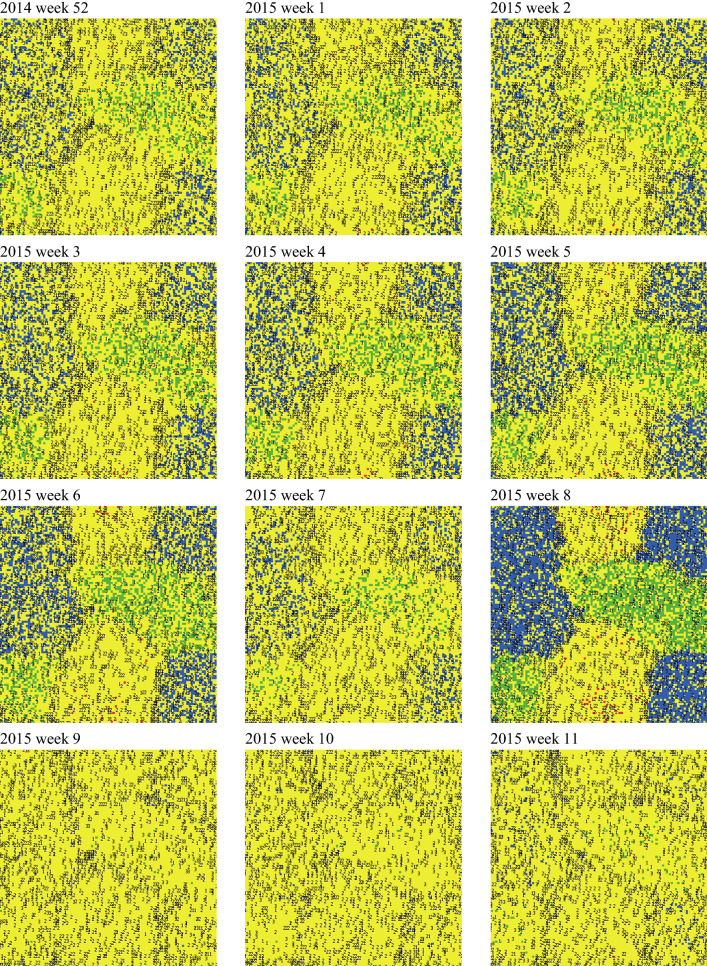
Time series of maps illustrating spatial dynamics of a tick infestation within the hypothetical 10,000-ha ranch containing good (green), fair (red), and poor (blue) tick habitat types. Acaricide applications capable of complete and continuous elimination of all on-host ticks applied to cattle and nilgai (but not white-tailed deer) were initiated during week 25 of 2009. Yellow represents infested landscape cells

The elimination of all on-host ticks on either foraging/grazing (i) cattle, or (ii) WTD, or (iii) nilgai had virtually no effect on the proportions of infested landscape patches in good, fair, and poor tick habitat types (Fig. 3). For all practical purposes, the entire ranch remained completely infested. The elimination of all on-host ticks on (iv) cattle and WTD, (v) WTD and nilgai, or (vi) cattle and nilgai had more noticeable effects (Fig. 4). Differences in proportions of infested landscape patches from year to year were due to primarily to differences in winter weather conditions, whereas infestation differences among tick habitat types were due primarily to habitat use preferences of hosts. The proportions of infested landscape patches occasionally dropped as low as approximately 0.6 when all on-host ticks were eliminated from cattle and WTD, or from WTD and nilgai. When all on-host ticks were eliminated from cattle and nilgai, the proportions of infested landscape patches periodically dropped below 0.4, occasionally dropping as low as 0.2, and infestation differences among tick habitat types were altered (Fig. 4). With WTD being the only distributor of ticks across the landscape, and cattle and nilgai acting to eliminate host-seeking larvae, the habitat use patterns of the hosts in effect created a refuge for off-host ticks in the fair tick habitat, particularly during periods of unfavorable conditions for off-host tick survival (Figs. 5, 6). An expanded time series of maps illustrating the appearance and disappearance of these refuges can be found in Additional file 1: Figure S15.

## Discussion

Over the last four decades, ungulate wildlife such as WTD and nilgai have adversely affected success of the CFTEP [7, 10, 19]. In 2010, the need to refine current SCFT tick models to include SCFT host interactions was identified as a key research goal for success of the eradication programme [12]. Our model is the first attempt to meet this knowledge gap by simulating relationships among nilgai, cattle and WTD. Other spatially-explicit, individual- or agent-based models simulating relationships among climate, landscape, hosts, and ticks include those of Estrada-Peña et al. [28] and Wang et al. [14, 31–33], which are reviewed by Wang et al. [26]. Only those of Estrada-Peña et al. [28] and Wang et al. [14] were focused on SCFT and included WTD as an alternate host. The model of Estrada-Peña et al. [28] represented movement of WTD based on graph theory and examined efficacy of anti-tick vaccination of WTD in controlling SCFT infestations using hypothetical vaccination schedules for individual animals. The model of Wang et al. [14], upon which the present model extension was based, examined potential effects of WTD on efficacy of three official SCFT eradication protocols. Model evaluation simulations suggested their model was capable of representing the complex interactions of weather patterns with host usage of different habitat types, which generated patterns of SCFT dispersal and subsequent off-host survivorship comparable to those documented by available field data [34]. Results of model eradication simulations were consistent with historic records of recrudescent infestations [10] and suggested that WTD may be capable of creating refugia for SCFT in space and time that could sustain tick populations and compromise eradication efforts involving cattle-centric approaches.

Our evaluation and confirmation of the present model [35], as a proof of concept to accommodate a third host-type, is based on (i) the robustness of general trends in model behavior to alterations of model structure and function, (ii) the sensitivity of the details of model behavior to changes in values of important model parameters, and (iii) the ability of the model to produce useful output. The model is both robust and sensitive [36]. That is, while clearly recognizable general patterns emerge, model behavior is appropriately responsive to changes in representation of important causal processes. For example, seasonal variation in simulated densities of off-host tick larvae generally follows a bimodal pattern, with a spring increase, summer decline, followed by autumn increase and winter decline (e.g. Additional file 1: Figure S3a). On the one hand, this trend is robust to changes in host species present, relative habitat preferences of hosts, and relative densities of hosts. On the other hand, specific magnitudes of mean weekly densities of off-host larvae are sensitive to (responsive to) specific changes in parameterization of each of these three factors. Analogously, seasonal variation in mean numbers of adult ticks per host, which generally remain relatively high during spring, summer and autumn, and decline to relatively low levels during winter (e.g. Additional file 1: Figure S3b, c and d), is robust to changes in the three host community attributes. Specific weekly mean numbers of adult ticks per host are sensitive to specific changes in parameterization of relative densities of hosts, but relatively insensitive to changes in parameterization of the other two host community attributes.

Regarding ability of the model to produce useful output, results of baseline simulations seem reasonable and are irrefutable based on available field data and observations, results of infestation simulations are interpretable ecologically, and results of eradication simulations have clear management implications with regard to the potential role of nilgai in sustaining SCFT populations. Not surprisingly, published data on abundances of both off- and on-host life stages of SCFT in south Texas rangelands are rare, in part due to quarantine regulations, difficulty of sampling the environment for off-host larval SCFT, and intricacies of sampling SCFT on wildlife hosts. Thus, assessment of reasonableness of quantitative aspects of simulation results must be guided primarily by comparative reasoning. For example, baseline simulations (i.e. with baseline densities and relative habitat preferences of cattle, WDT and nilgai; Additional file 1: Table S1) generate annual variations in abundance of host-seeking larvae (following the bimodal pattern described in the previous paragraph) which are similar to those observed in New Caledonia [37]. Simulated maximum numbers of adult ticks on cattle, WTD and nilgai are ≈ 50, ≈ 4.5 and ≈ 25, respectively. Bourne et al. [38] reported that *Bos taurus* cattle in central and southern Queensland carried an average of 465 and 302 standard-sized female SCFT, respectively. Currie [39] reported an average infestation of 13 adult SCFT per WTD in south Texas (Zapata County). Olafson et al. [19] reported a mean of 5.4 SCFT per nilgai in south Texas (Cameron and Willacy counties). The relative metabolic body size and agility of nilgai indicate they are as ill-equipped as cattle to remove ticks by grooming, suggesting that 5.4 may be at the low end of probable per capita tick loads. Thus, simulated tick loads on all hosts most likely are conservative, which is appropriate given our problem context.

Within a management context, eradication simulations suggest that nilgai do, indeed, have the potential to sustain SCFT populations, even in the absence of other hosts. It appears the role of nilgai in sustaining SCFT infestations may be complimentary, rather than strictly analogous, to the role of WTD. Overlapping habitat use (tick deposition) patterns of these hosts may create refugia for SCFT during periods of unfavorable conditions for off-host tick survival, and facilitate local spread from these refugia during more favorable periods. Nilgai habitat use patterns may greatly facilitate the widespread redistribution and maintenance of SCFT during more favorable periods. Activity ranges of nilgai are almost an order of magnitude greater than those of WTD, with the maximum axis of the home ranges of radio-tracked individuals exceeding 30 km [21]. Additionally, male nilgai are capable of traversing their entire home range in a single day [40]. Tick population genetic analyses have confirmed shared local tick infestations on cattle and WTD, and that WTD spread SCFT more widely than cattle, excluding those cattle moved long distances by humans [41]. Although yet to be confirmed, it seems reasonable to suspect that local tick infestations are shared among cattle, WTD and nilgai, and that nilgai spread SCFT even more widely than WTD.

Modeling efforts, such as the present one, in coordination with field studies on interactions among cattle, WDT, nilgai and other potential host species, are crucial for development of integrated strategies for sustainable SCFT eradication in the USA [6]. Building on the work of Wang et al. [14], the present SCFT model provides (i) ability to hypothesize cause-effect relationships among host species, (ii) test capability of the hypothesized effects to produce patterns of tick abundance observed in the field, and (iii) simulate how these patterns could be altered by specific interventions in space and time, and in the tick life-cycle. Our eradication simulations, for example, tested the hypothesis that cattle could function as “sponges” to “trap” and remove SCFT in order to sustain eradication in persistently-infested premises co-inhabited by cattle and WTD [12] (and nilgai). Simulation of spatially-explicit chronologies of shifts in off- and on-host densities of SCFT also could suggest when and where monitoring and treatment efforts might be most effective. This would allow design and a priori testing of field sampling strategies in particular landscapes under specific conditions [14]. Thus, the present model should provide a useful tool to aid in prioritization of integrated efforts to suppress SCFT populations in the USA.

## Conclusions

Infestations in nilgai augment SCFT refugia enabled by WTD and promote pest persistence across the landscape and cattle parasitism. Our study demonstrated the utility of enhanced biosurveillance using simulation tools to mitigate risk and enhance operations of area-wide tick management programmes like the CFTEP through integrated tactics for SCFT suppression.

## Supplementary information

**Additional file 1: Figure S1.** Map of historical infestations of the southern cattle fever tick. **Figure S2.** Weather profiles. **Figure S3.** Assessment of host contribution. **Figure S4.** Assessment of habitat usage. **Figure S5.** Simulated mean numbers of off-host tick larvae per hectare from January 2009 through December 2018. **Figure S6.** Assessment of host contribution in three habitats from January 2014 through December 2015. **Figure S7.** Assessment of host density in three habitats in December 2014. **Figure S8.** Assessment of host density on numbers of off-host ticks and adult ticks on hosts in December 2014. **Figure S9.** Assessment of single host contribution on numbers of off-host ticks in December 2014. **Figure S10.** Assessment of single host contribution on numbers of adult ticks on hosts in December 2014. **Figure S11.** Assessment of contribution of single infested host introduced in the middle of a patch of fair tick habitat from June 2009 through January 2010. **Figure S12.** Assessment of contribution of single infested host introduced in the middle of a patch of good tick habitat from June 2009 through January 2010. **Figure S13.** Assessment of contribution of single infested host introduced in the middle of a patch of poor tick habitat from June 2009 through January 2010. **Figure S14.** Time series of maps illustrating spatial spread of a tick infestation with one infested head of cattle introduced in June 2009. **Figure S15.** Time series of maps illustrating spatial dynamics of a tick infestation with acaricide applications initiated in June 2009. **Table S1.** List of the parameters used to represent nilgai, cattle, and white-tailed deer as hosts of cattle fever ticks, their baseline values, and their information sources.

## Data Availability

The simulated data during and/or analyzed during the present study are available from the corresponding author upon reasonable request.

## Abbreviations

CFT: Cattle fever tick
SCFT: Southern cattle fever tick
PQZ: Permanent Quarantine Zone
WTD: White-tailed deer
PCR: Polymerase chain reaction
USDA: United States Department of Agriculture
APHIS: Animal Plant Health and Inspection Service
