# Supplementary material for: Enhanced biosurveillance of high-consequence invasive pests: southern cattle fever ticks, Rhipicephalus (Boophilus) microplus, on livestock and wildlife
Source: Parasit Vectors. 2020 Sep 23;13:487. doi: 10.1186/s13071-020-04366-x (PMC7513513; doi:10.1186/s13071-020-04366-x)
Supplement: Supplementary file 1 — Additional file 1: Figure S1. Map of historical infestations of the southern cattle fever tick. Figure S2. Weather profiles. Figure S3. Assessment of host contribution. Figure S4. Assessment of habitat usage. Figure S5. Simulated mean numbers of off-host tick larvae per hectare from January 2009 through December 2018. Figure S6. Assessment of host contribution in three habitats from January 2014 through December 2015. Figure S7. Assessment of host density in three habitats in December 2014. Figure S8. Assessment of host density on numbers of off-host ticks and adult ticks on hosts in December 2014. Figure S9. Assessment of single host contribution on numbers of off-host ticks in December 2014. Figure S10. Assessment of single host contribution on numbers of adult ticks on hosts in December 2014. Figure S11. Assessment of contribution of single infested host introduced in the middle of a patch of fair tick habitat from June 2009 through January 2010. Figure S12. Assessment of contribution of single infested host introduced in the middle of a patch of good tick habitat from June 2009 through January 2010. Figure S13. Assessment of contribution of single infested host introduced in the middle of a patch of poor tick habitat from June 2009 through January 2010. Figure S14. Time series of maps illustrating spatial spread of a tick infestation with one infested head of cattle introduced in June 2009. Figure S15. Time series of maps illustrating spatial dynamics of a tick infestation with acaricide applications initiated in June 2009. Table S1. List of the parameters used to represent nilgai, cattle, and white-tailed deer as hosts of cattle fever ticks, their baseline values, and their information sources. [file 13071_2020_4366_MOESM1_ESM.pdf]

Additional file 1

Supplementary Information for Enhanced Biosurveillance of High-Consequence Invasive Pests: Cattle Fever Ticks, *Rhipicephalus* (*Boophilus*) *microplus*, on Livestock and Wildlife

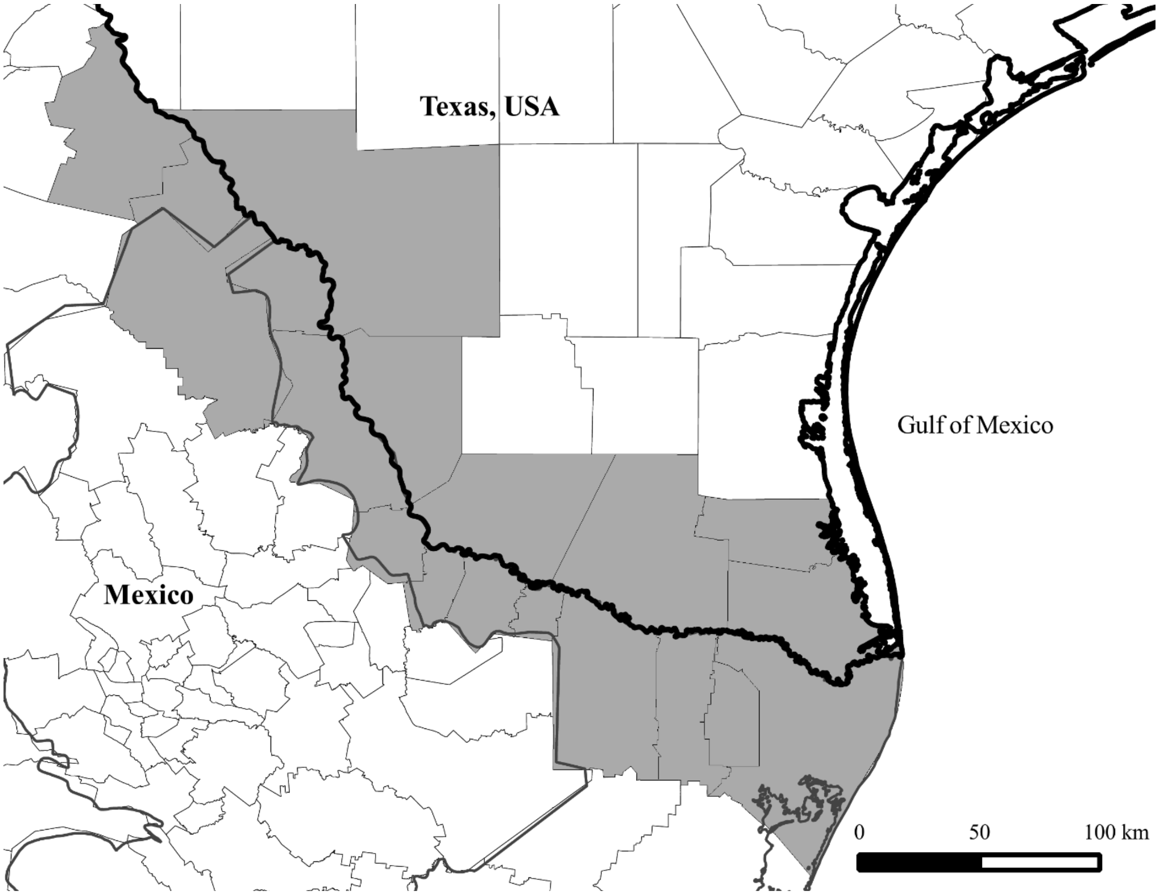

Figure S1 Map of adjoining principalities along the transboundary region of Texas, USA/Mexico with historical infestations of the southern cattle fever tick (*Rhipicephalus* (*Boophilus*) *microplus*)

a

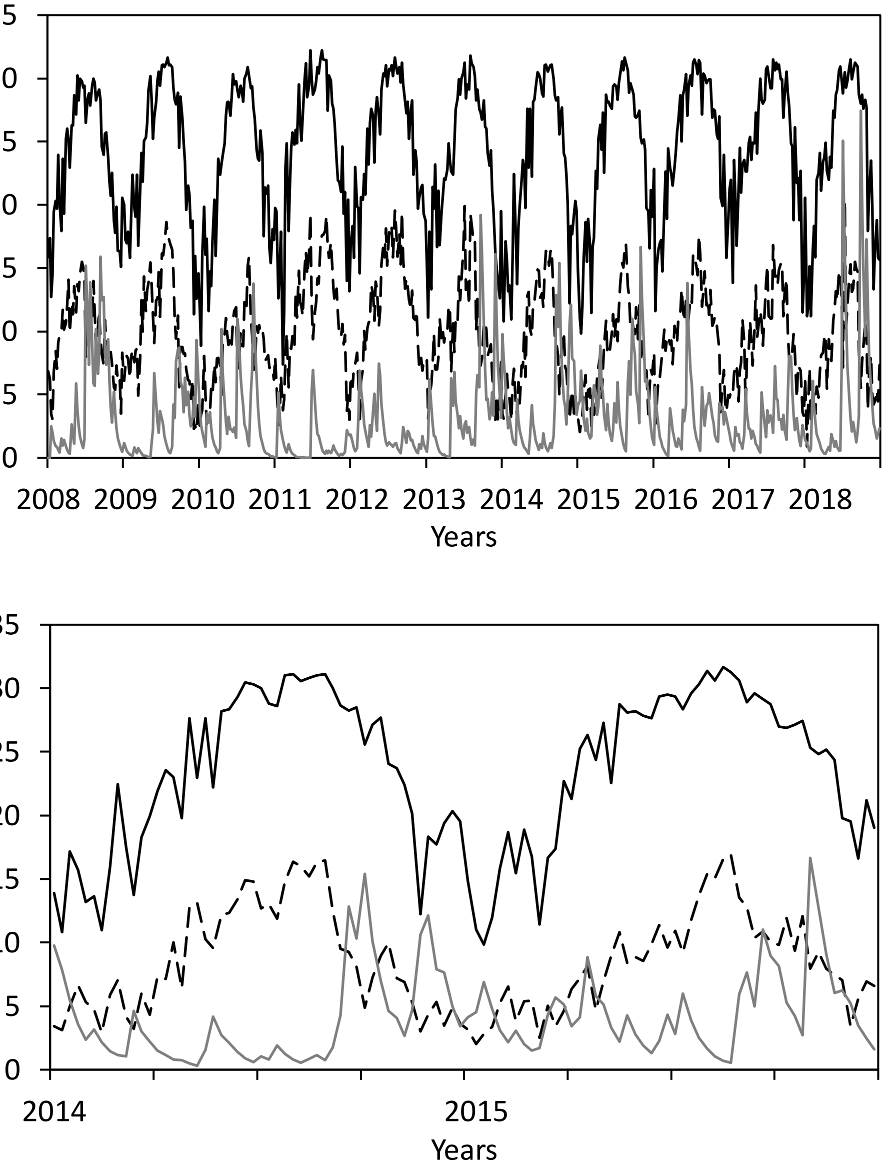

Figure S2 Weather profiles. Weekly air temperatures (°C, black line), saturation deficits (millibars, dash line), and index values based on precipitation (cm, grey line) recorded in Willacy County, Texas, USA (**a**) from January 2008 through December 2018 and (**b**) from January 2014 through December 2015

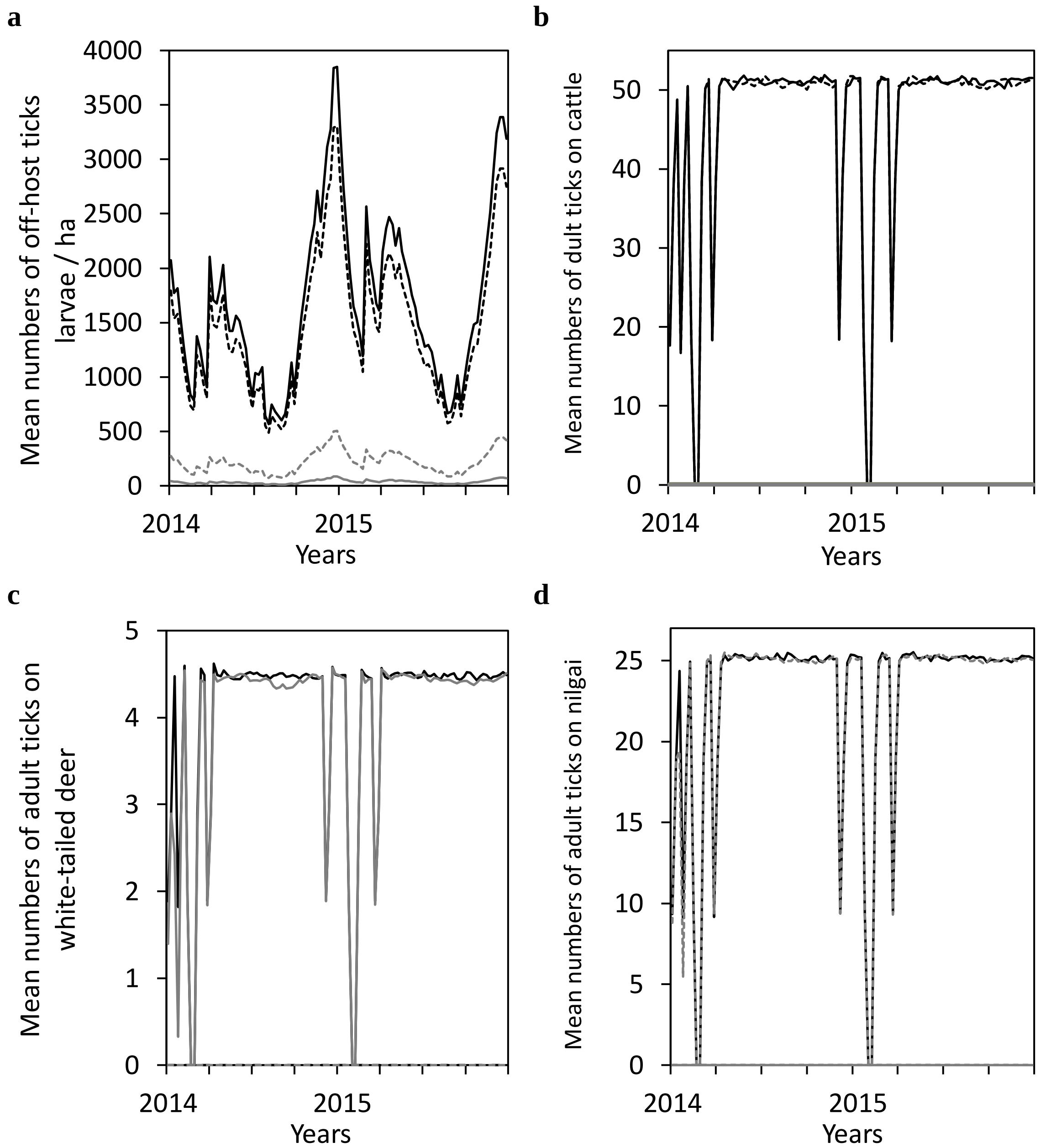

Figure S3 Assessment of host contribution. Simulated mean numbers of (a) off-host (potentially host-seeking) tick larvae per hectare, and adult ticks on (b) cattle, (c) white-tailed deer, and (d) nilgai on a hypothetical 10,000-hectare ranch under weather conditions recorded in Willacy County, Texas, USA from January 2009 through December 2018 (only January 2014 through December 2015 shown here). Simulations assumed tick hosts present on the ranch included (1) cattle, white-tailed deer, and nilgai (black line), (2) only cattle (black dash line), (3) only white-tailed deer (grey line), and (4) only nilgai (grey dash line). Thirty-one percent, 28 percent, and 41 percent of the ranch was considered good, fair, and poor habitat, respectively, for off-host tick larvae. Relative habitat use preferences of hosts for good, fair, and poor tick habitats, respectively, were 0.30, 0.10, and 0.60 for cattle, 0.20, 0.40, and 0.40 for white-tailed deer, and 0.30, 0.10, and 0.60 for nilgai

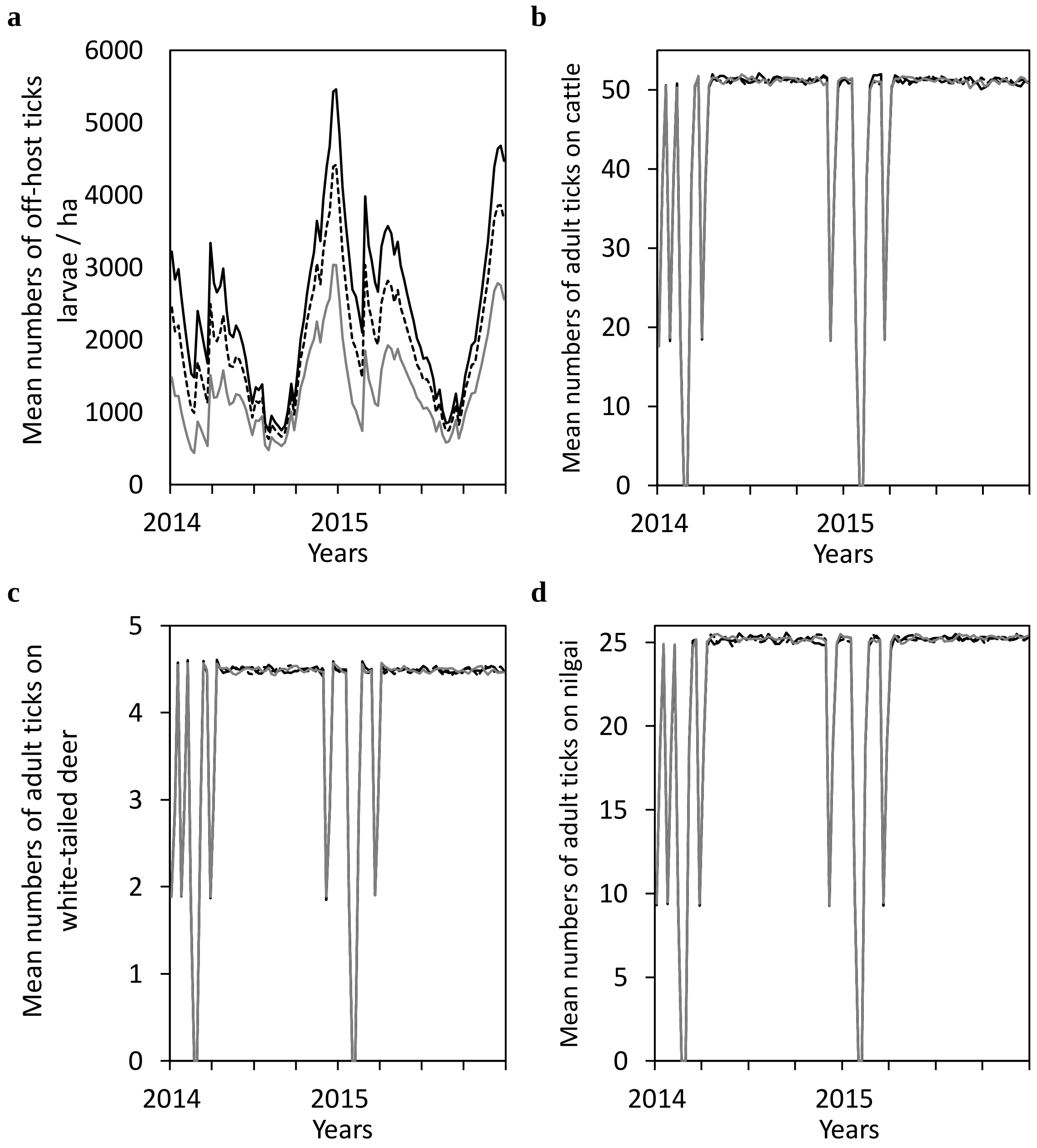

Figure S4 Assessment of habitat usage. Simulated mean numbers of (**a**) off-host (potentially host-seeking) tick larvae per hectare, and adult ticks on (**b**) cattle, (**c**) white-tailed deer, and (**d**) nilgai on a hypothetical 10,000-hectare ranch under weather conditions recorded in Willacy County, Texas, USA from January 2009 through December 2018 (only January 2014 through December 2015 shown here). Simulations assumed relative habitat use preferences of all hosts for good, fair, and poor tick habitats, respectively, were (1) 1, 0, and 0 (black line), (2) 0, 1, and 0 (dash line), and (3) 0, 0, and 1 (grey line). Tick hosts present on the ranch included cattle, white-tailed deer, and nilgai. Thirty-one percent, 28 percent, and 41 percent of the ranch was considered good, fair, and poor habitat, respectively, for off-host tick larvae

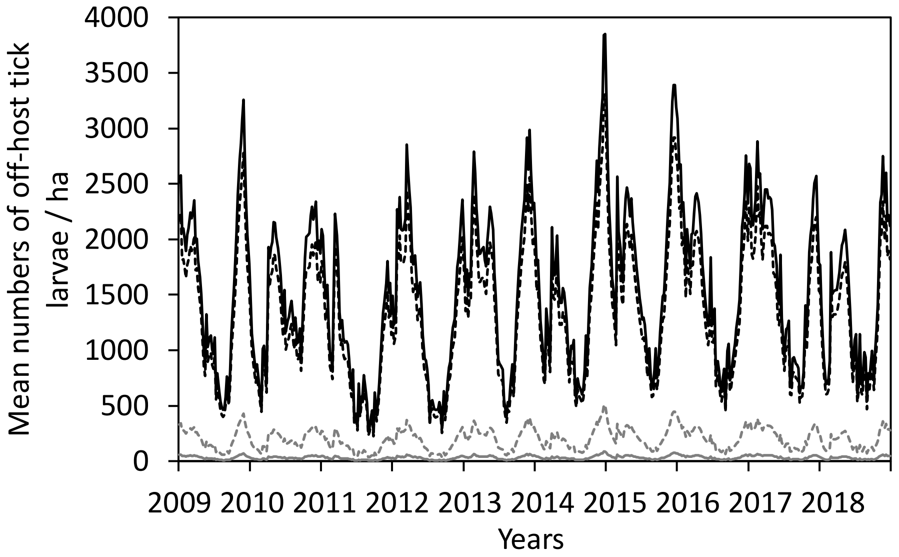

Figure S5 Simulated mean numbers of off-host (potentially host-seeking) tick larvae per hectare on a hypothetical 10,000-hectare ranch under weather conditions recorded in Willacy County, Texas, USA from January 2009 through December 2018. Simulations assumed tick hosts present on the ranch included (1) cattle, white-tailed deer, and nilgai (black line), (2) only cattle (black dash line), (3) only white-tailed deer (grey line), and (4) only nilgai (grey dash line). Thirty-one percent, 28 percent, and 41 percent of the ranch was considered good, fair, and poor habitat, respectively, for off-host tick larvae. Relative habitat use preferences of hosts for good, fair, and poor tick habitats, respectively, were 0.30, 0.10, and 0.60 for cattle, 0.20, 0.40, and 0.40 for white-tailed deer, and 0.30, 0.10, and 0.60 for nilgai

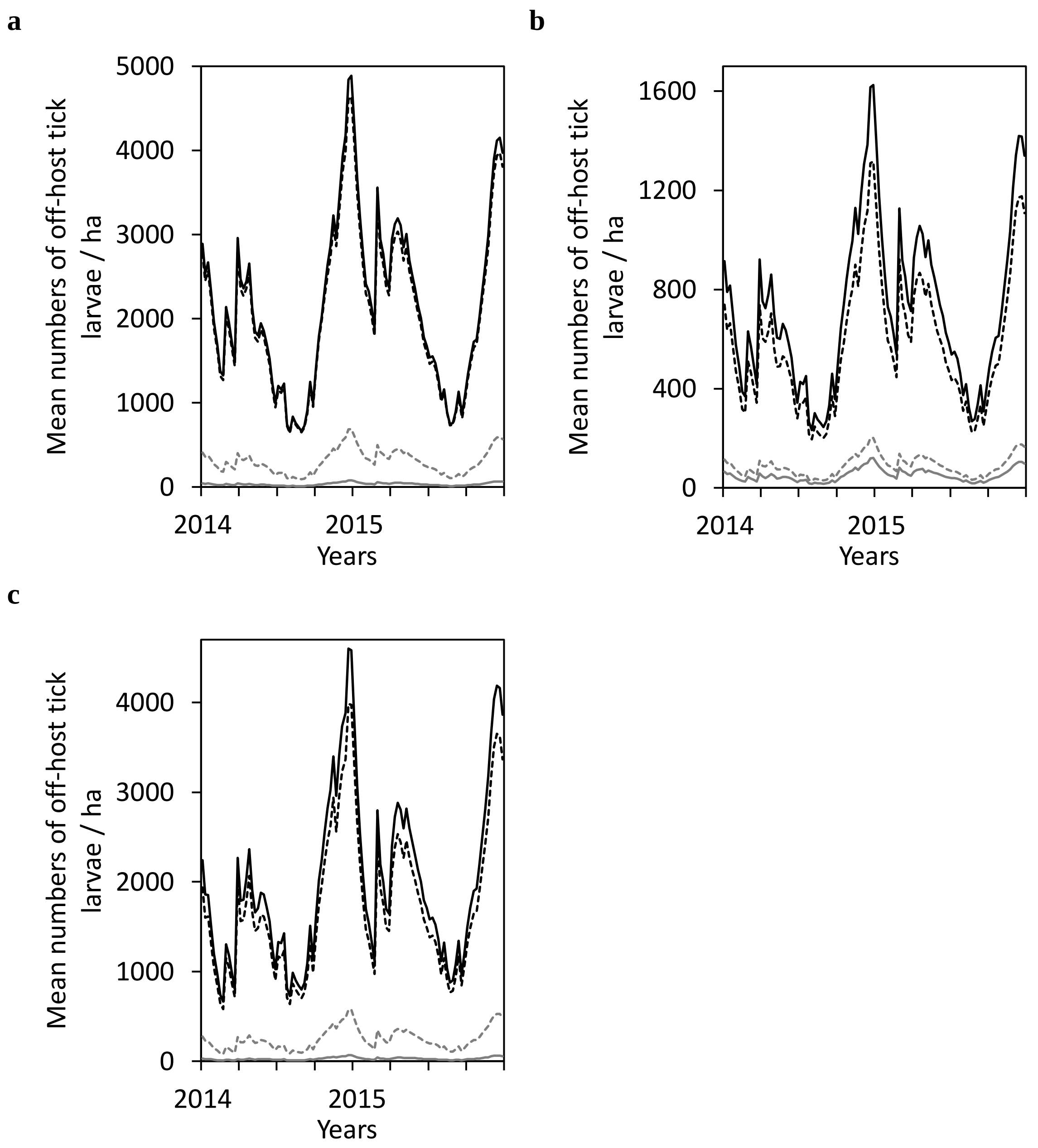

Figure S6 Simulated mean numbers of off-host (potentially host-seeking) tick larvae per hectare in (**a**) good, (**b**) fair, and (**c**) poor tick habitat on a hypothetical 10,000-hectare ranch under weather conditions recorded in Willacy County, Texas, USA from January 2014 through December 2015. Simulations assumed tick hosts present on the ranch included (1) cattle, white-tailed deer, and nilgai (black line), (2) only cattle (black dash line), (3) only white-tailed deer (grey line), and (4) only nilgai (grey dash line). Thirty-one percent, 28 percent, and 41 percent of the ranch was considered good, fair, and poor habitat, respectively, for off-host tick larvae. Relative habitat use preferences of hosts for good, fair, and poor tick habitats, respectively, were 0.30, 0.10, and 0.60 for cattle, 0.20, 0.40, and 0.40 for white-tailed deer, and 0.30, 0.10, and 0.60 for nilgai

a.

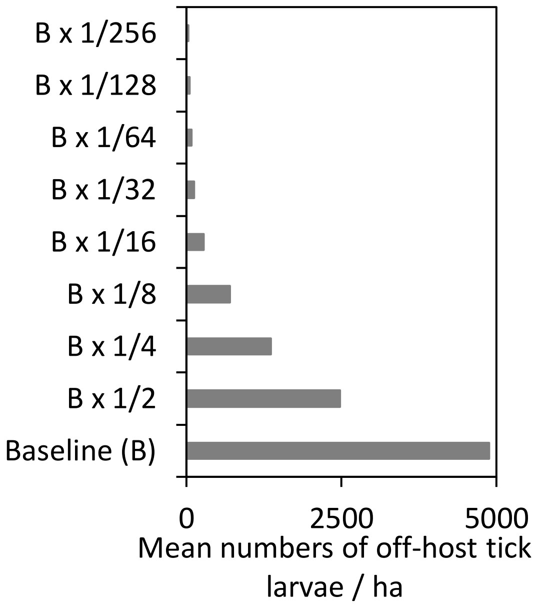

b.

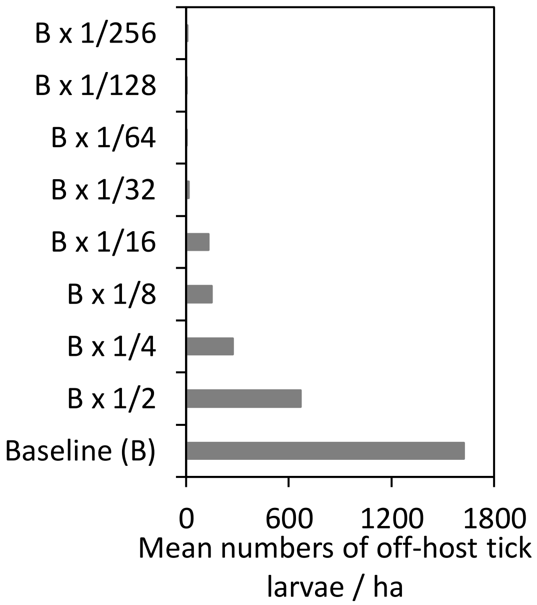

c.

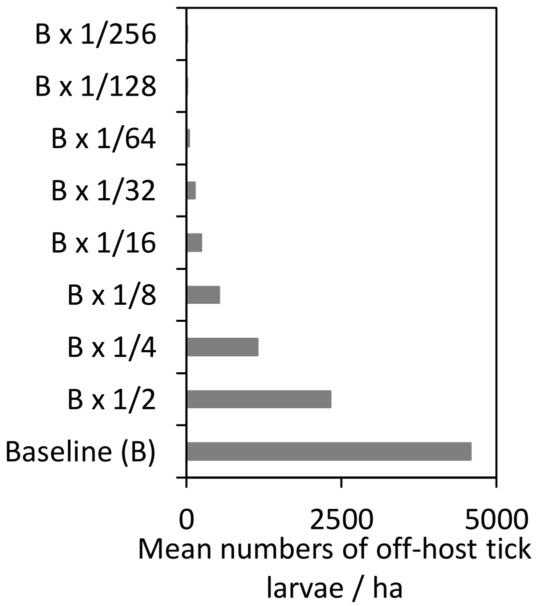

Figure S7 Simulated mean numbers of off-host (potentially host-seeking) tick larvae per hectare in (**a**) good, (**b**) fair, and (**c**) poor tick habitat on a hypothetical 10,000-hectare ranch under weather conditions recorded in Willacy County, Texas, USA during the last week in December, 2014. Simulations assumed densities of all host species were at (1) baseline levels, as well as at (2) 1/2, (3) 1/4, (4) 1/8, (5) 1/16, (6) 1/32, (7) 1/64, (8) 1/128, and (9) 1/256 of baseline levels (Suppl. Inf., Table S1). Tick hosts present on the ranch included cattle, white-tailed deer, and nilgai. Thirty-one percent, 28 percent, and 41 percent of the ranch was considered good, fair, and poor habitat, respectively, for off-host tick larvae. Relative habitat use preferences of hosts for good, fair, and poor tick habitats, respectively, were 0.30, 0.10, and 0.60 for cattle, 0.20, 0.40, and 0.40 for white-tailed deer, and 0.30, 0.10, and 0.60 for nilgai

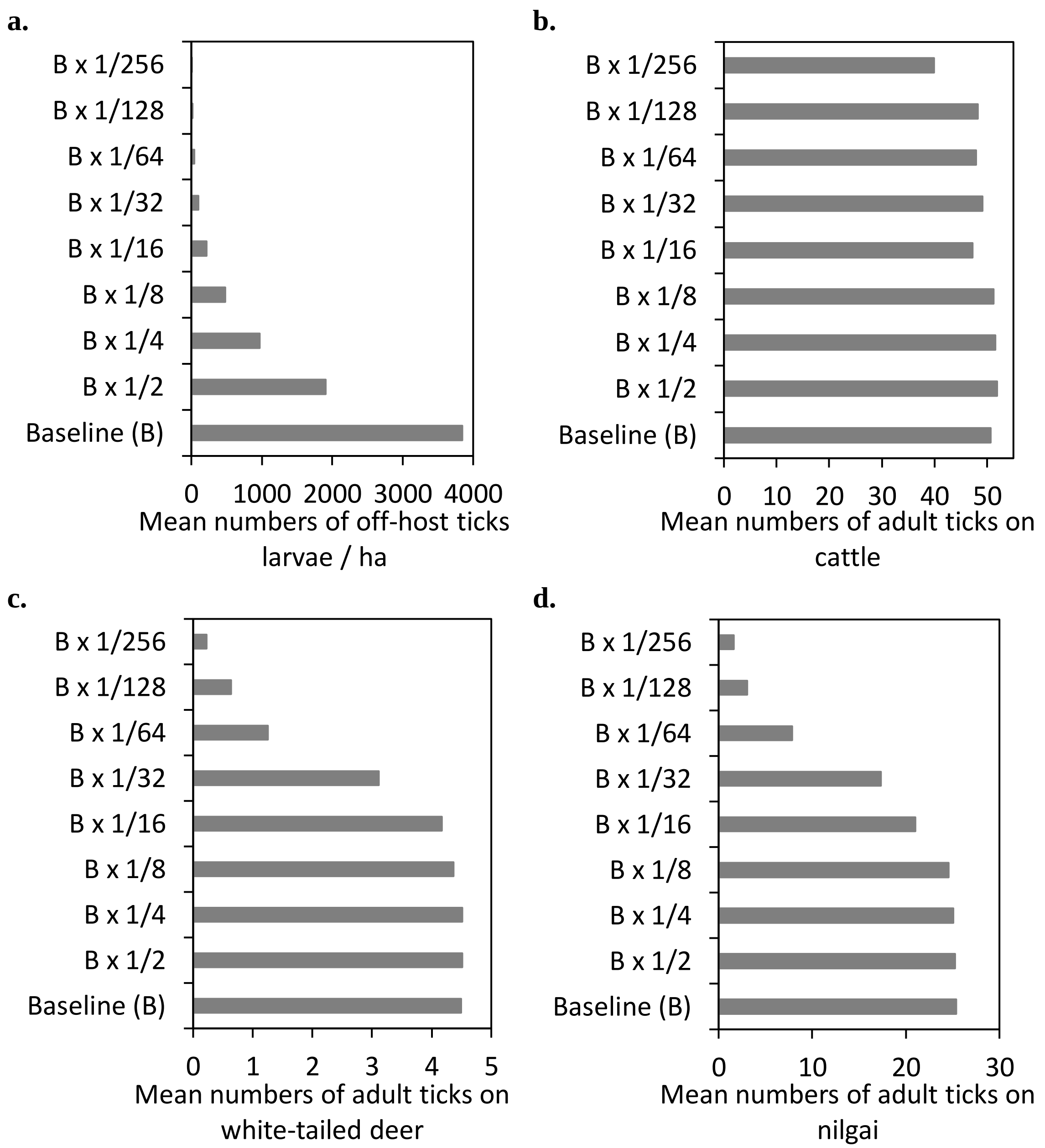

Figure S8 Assessment of host density. Simulated mean numbers of (**a**) off-host (potentially host-seeking) tick larvae per hectare, and adult ticks on (**b**) cattle, (**c**) white-tailed deer, and (**d**) nilgai on a hypothetical 10,000-hectare ranch under weather conditions recorded in Willacy County, Texas, USA during the last week in December, 2014. Simulations assumed densities of all host species were at (1) baseline levels, as well as at (2) 1/2, (3) 1/4, (4) 1/8, (5) 1/16, (6) 1/32, (7) 1/64, (8) 1/128, and (9) 1/256 of baseline levels (Suppl. Inf., Table S1). Tick hosts present on the ranch included cattle, white-tailed deer, and nilgai. Thirty-one percent, 28 percent, and 41 percent of the ranch was considered good, fair, and poor habitat, respectively, for off-host tick larvae. Relative habitat use preferences of hosts for good, fair, and poor tick habitats, respectively, were 0.30, 0.10, and 0.60 for cattle, 0.20, 0.40, and 0.40 for white-tailed deer, and 0.30, 0.10, and 0.60 for nilgai

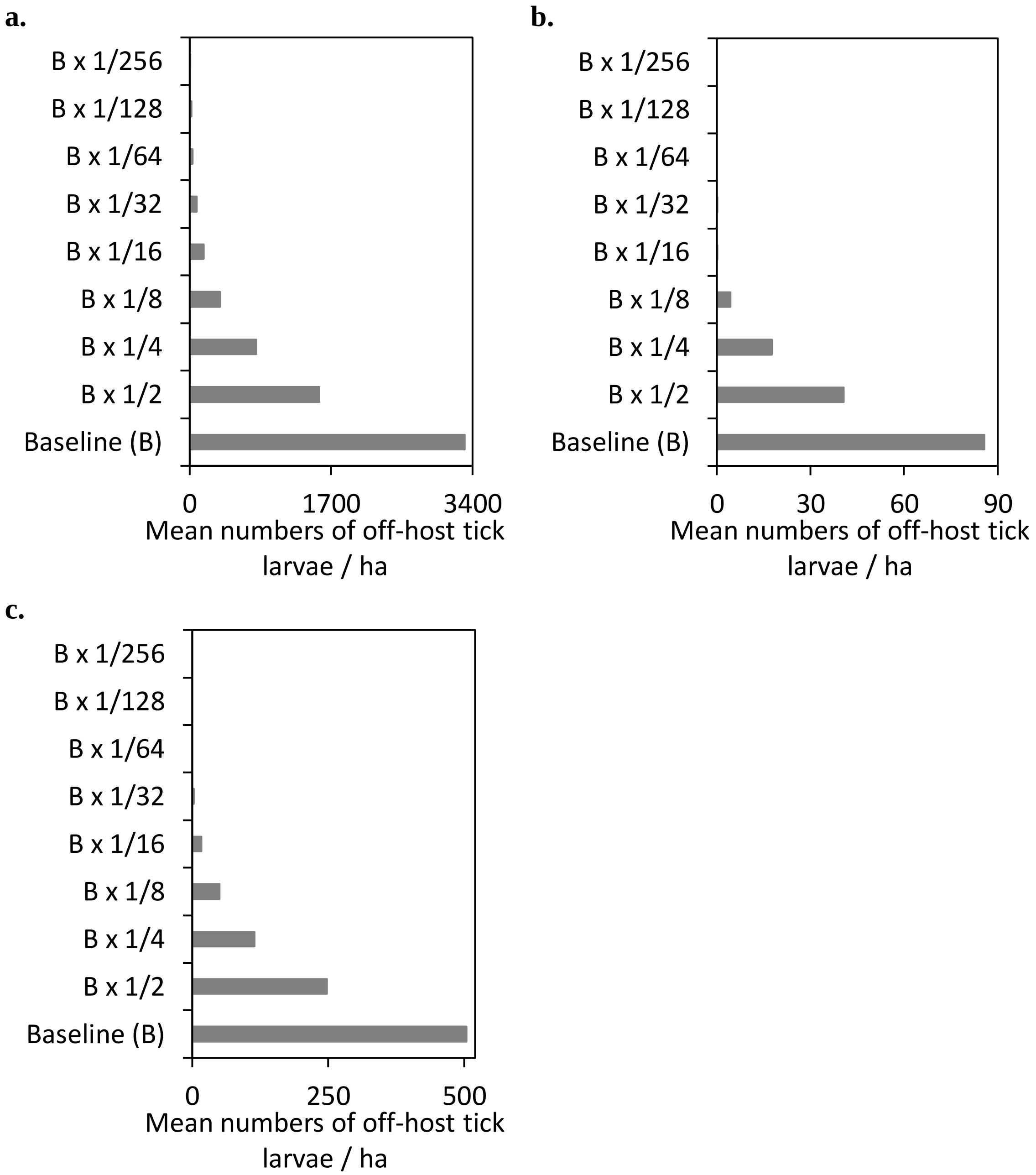

Figure S9 Simulated mean numbers of off-host (potentially host-seeking) tick larvae per hectare on a hypothetical 10,000-hectare ranch under weather conditions recorded in Willacy County, Texas, USA during the last week in December, 2014. Simulations assumed densities of cattle, white-tailed deer, and nilgai were at (1) baseline levels, as well as at (2) 1/2, (3) 1/4, (4) 1/8, (5) 1/16, (6) 1/32, (7) 1/64, (8) 1/128, and (9) 1/256 of baseline levels (Suppl. Inf., Table S1). Tick hosts present on the ranch included only (**a**) cattle, (**b**) white-tailed deer, or (**c**) nilgai. Thirty-one percent, 28 percent, and 41 percent of the ranch was considered good, fair, and poor habitat, respectively, for off-host tick larvae. Relative habitat use preferences of hosts for good, fair, and poor tick habitats, respectively, were 0.30, 0.10, and 0.60 for cattle, 0.20, 0.40, and 0.40 for white-tailed deer, and 0.30, 0.10, and 0.60 for nilgai

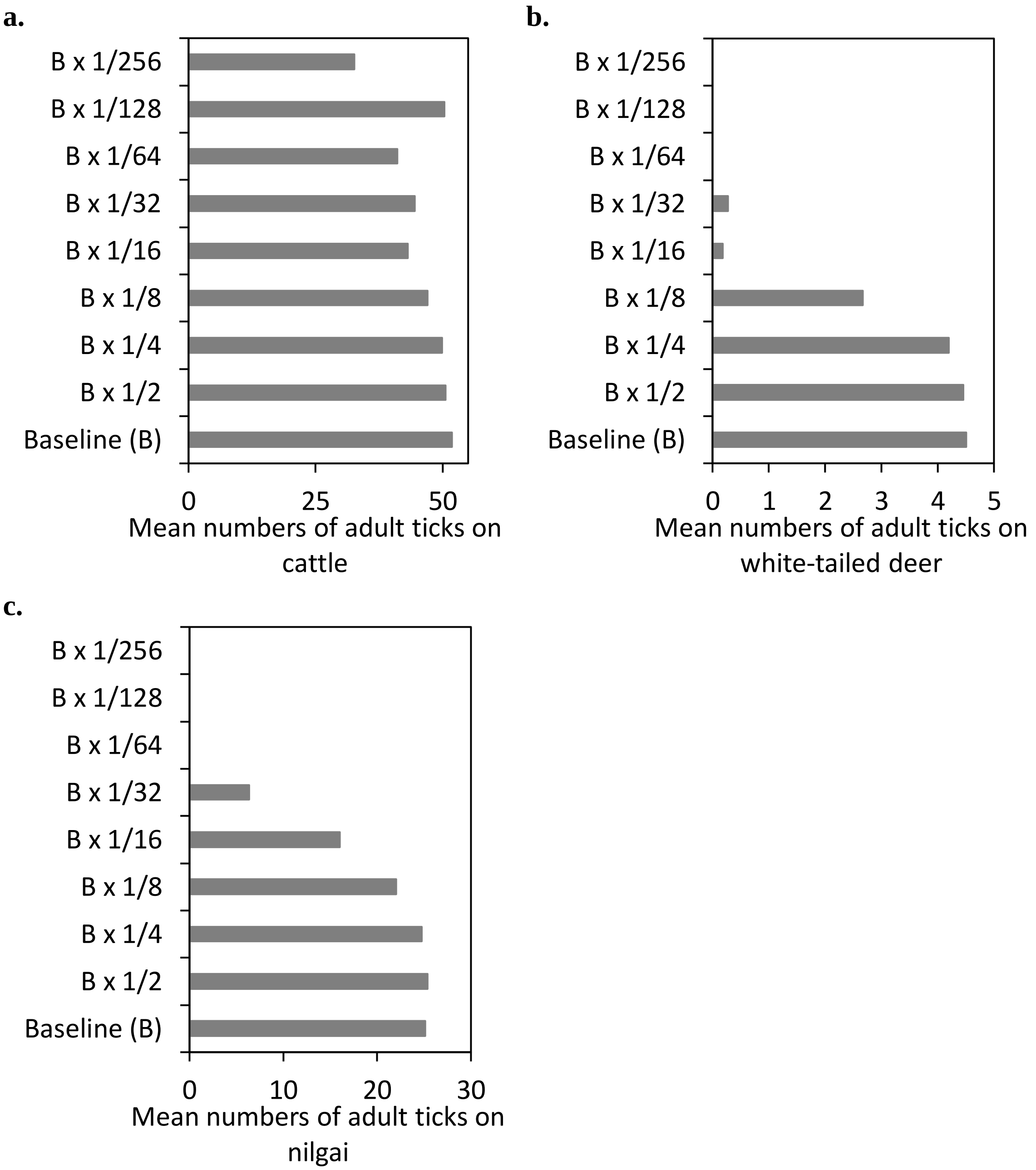

Figure S10 Simulated mean numbers of adult ticks on cattle, white-tailed deer, and nilgai on a hypothetical 10,000-hectare ranch under weather conditions recorded in Willacy County, Texas, USA during the last week in December, 2014. Simulations assumed densities of cattle were at (1) baseline levels, as well as at (2) 1/2, (3) 1/4, (4) 1/8, (5) 1/16, (6) 1/32, (7) 1/64, (8) 1/128, and (9) 1/256 of baseline levels (Suppl. Inf., Table S1). Tick hosts present on the ranch included only (**a**) cattle, (**b**) white-tailed deer, or (**c**) nilgai. Thirty-one percent, 28 percent, and 41 percent of the ranch was considered good, fair, and poor habitat, respectively, for off-host tick larvae. Relative habitat use preferences of hosts for good, fair, and poor tick habitats, respectively, were 0.30, 0.10, and 0.60 for cattle, 0.20, 0.40, and 0.40 for white-tailed deer, and 0.30, 0.10, and 0.60 for nilgai

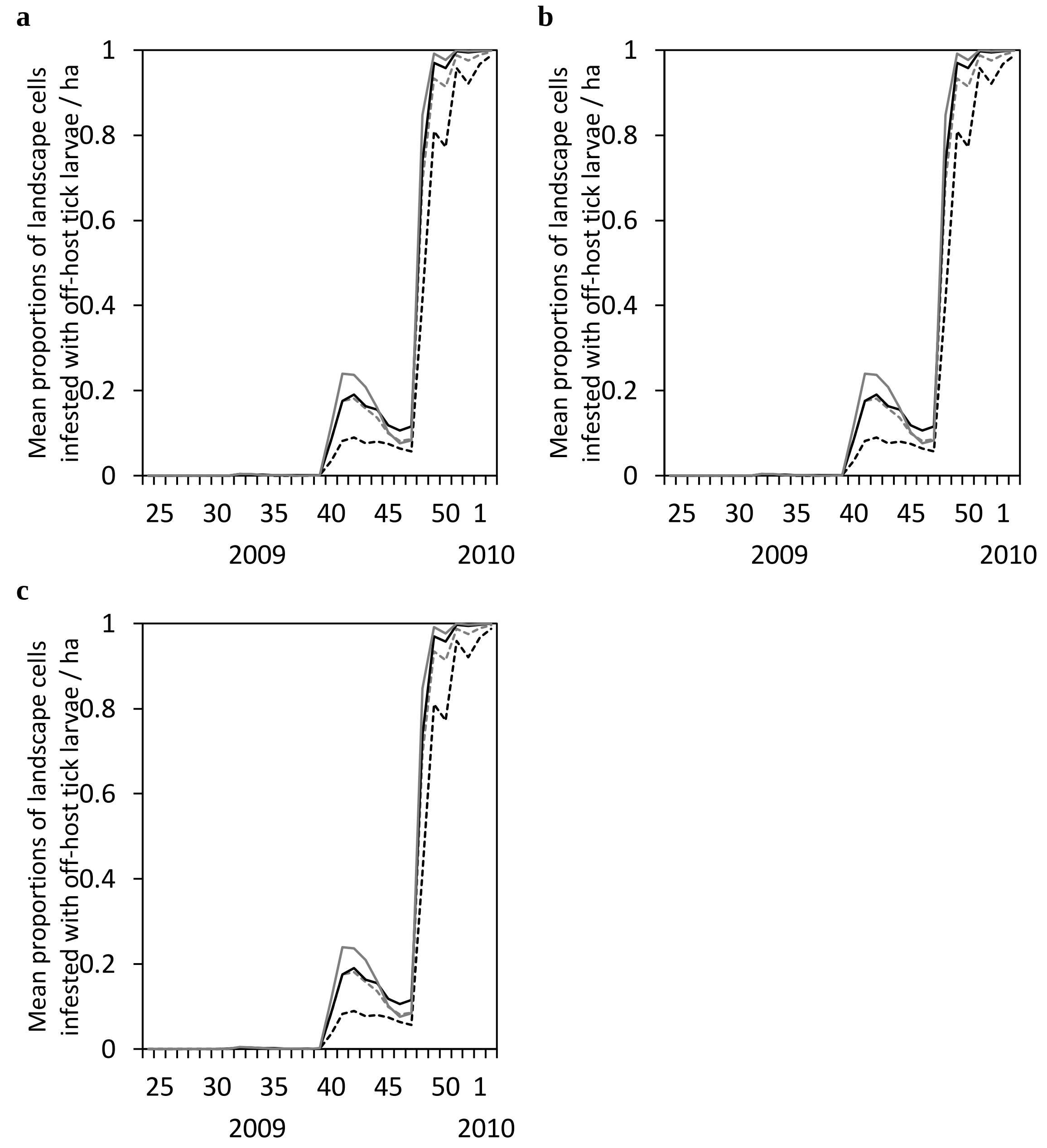

Figure S11 Simulated mean proportions of landscape cells infested with off-host (potentially host-seeking) tick larvae per hectare on a hypothetical 10,000-hectare ranch under weather conditions recorded in Willacy County, Texas, USA from January 2009 through December 2018 (only week 24, 2009 through week 2, 2010 shown here): (1) on whole ranch (grey dash line), and in (2) good (black line), (3) fair (black dash line), and (4) poor (grey line) tick habitats. Simulations assumed one infested (**a**) head of cattle, (**b**) white-tailed deer, or (**c**) nilgai was introduced in the middle of a patch of fair tick habitat in an otherwise CFT-free ranch during week 25 of 2009. Tick hosts present on the ranch included cattle, white-tailed deer, and nilgai. Thirty-one percent, 28 percent, and 41 percent of the ranch was considered good, fair, and poor habitat, respectively, for off-host tick larvae. Relative habitat use preferences of hosts for good, fair, and poor tick habitats, respectively, were 0.30, 0.10, and 0.60 for cattle, 0.20, 0.40, and 0.40 for white-tailed deer, and 0.30, 0.10, and 0.60 for nilgai

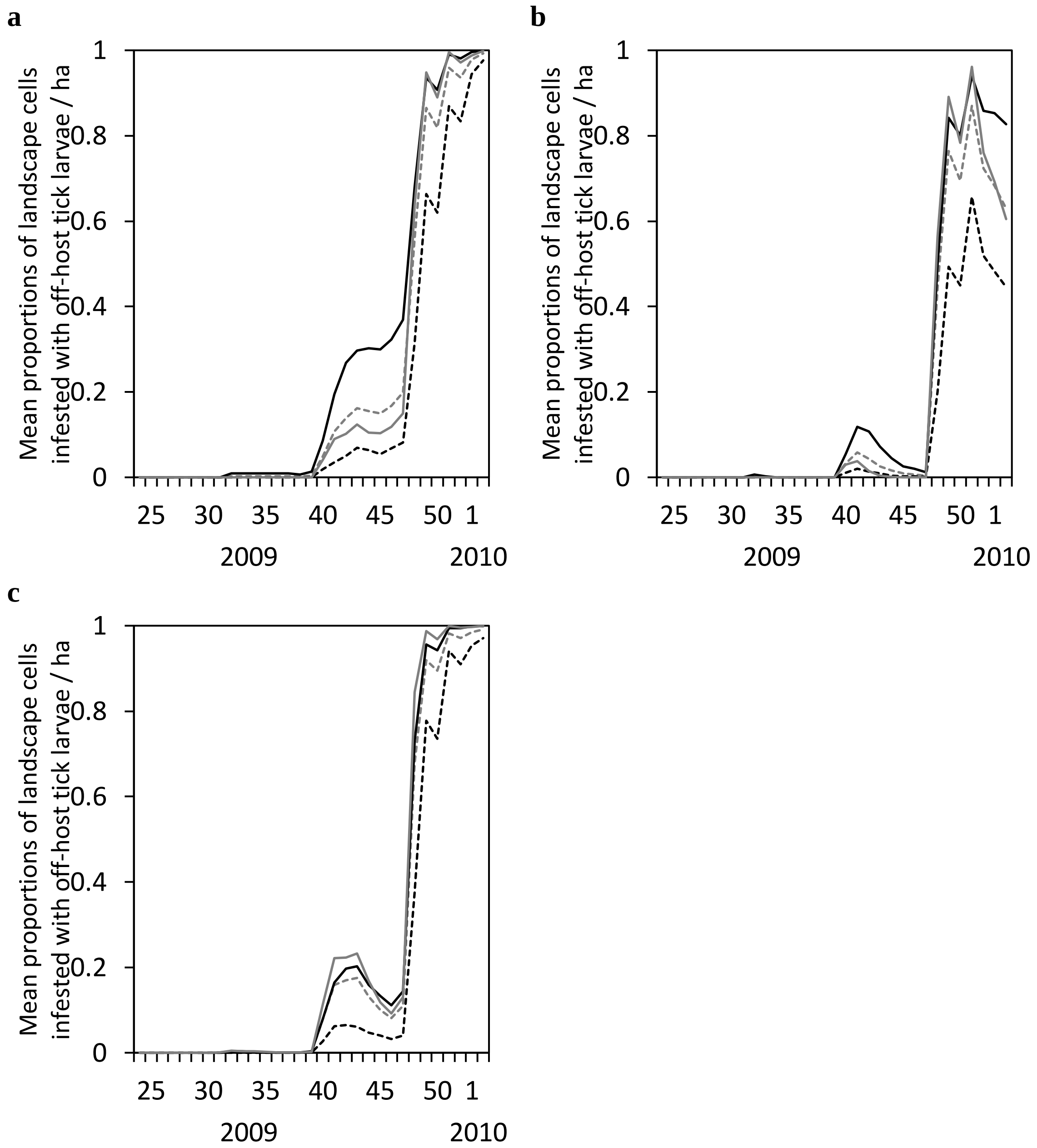

Figure S12 Simulated mean proportions of landscape cells infested with off-host (potentially host-seeking) tick larvae per hectare on a hypothetical 10,000-hectare ranch under weather conditions recorded in Willacy County, Texas, USA from January 2009 through December 2018 (only week 24, 2009 through week 2, 2010 shown here): (1) on whole ranch (grey dash line), and in (2) good (black line), (3) fair (black dash line), and (4) poor (grey line) tick habitats. Simulations assumed one infested (**a**) head of cattle, (**b**) white-tailed deer, or (**c**) nilgai was introduced in the middle of a patch of good tick habitat in an otherwise CFT-free ranch during week 25 of 2009. Tick hosts present on the ranch included cattle, white-tailed deer, and nilgai. Thirty-one percent, 28 percent, and 41 percent of the ranch was considered good, fair, and poor habitat, respectively, for off-host tick larvae. Relative habitat use preferences of hosts for good, fair, and poor tick habitats, respectively, were 0.30, 0.10, and 0.60 for cattle, 0.20, 0.40, and 0.40 for white-tailed deer, and 0.30, 0.10, and 0.60 for nilgai

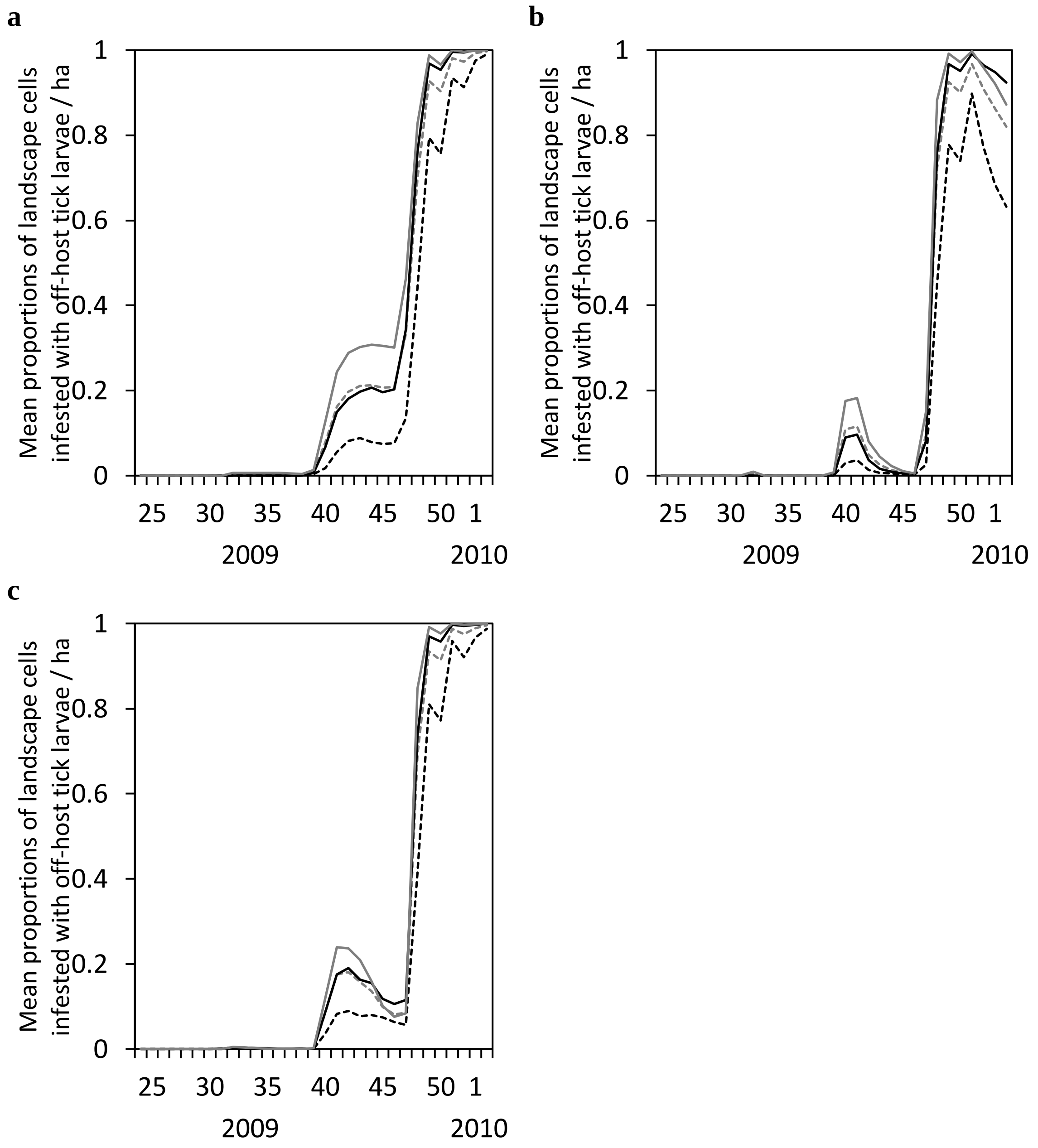

Figure S13 Simulated mean proportions of landscape cells infested with off-host (potentially host-seeking) tick larvae per hectare on a hypothetical 10,000-hectare ranch under weather conditions recorded in Willacy County, Texas, USA from January 2009 through December 2018 (only week 24, 2009 through week 2, 2010 shown here): (1) on whole ranch (grey dash line), and in (2) good (black line), (3) fair (black dash line), and (4) poor (grey line) tick habitats. Simulations assumed one infested (**a**) head of cattle, (**b**) white-tailed deer, or (**c**) nilgai was introduced in the middle of a patch of poor tick habitat in an otherwise CFT-free ranch during week 25 of 2009. Tick hosts present on the ranch included cattle, white-tailed deer, and nilgai. Thirty-one percent, 28 percent, and 41 percent of the ranch was considered good, fair, and poor habitat, respectively, for off-host tick larvae. Relative habitat use preferences of hosts for good, fair, and poor tick habitats, respectively, were 0.30, 0.10, and 0.60 for cattle, 0.20, 0.40, and 0.40 for white-tailed deer, and 0.30, 0.10, and 0.60 for nilgai

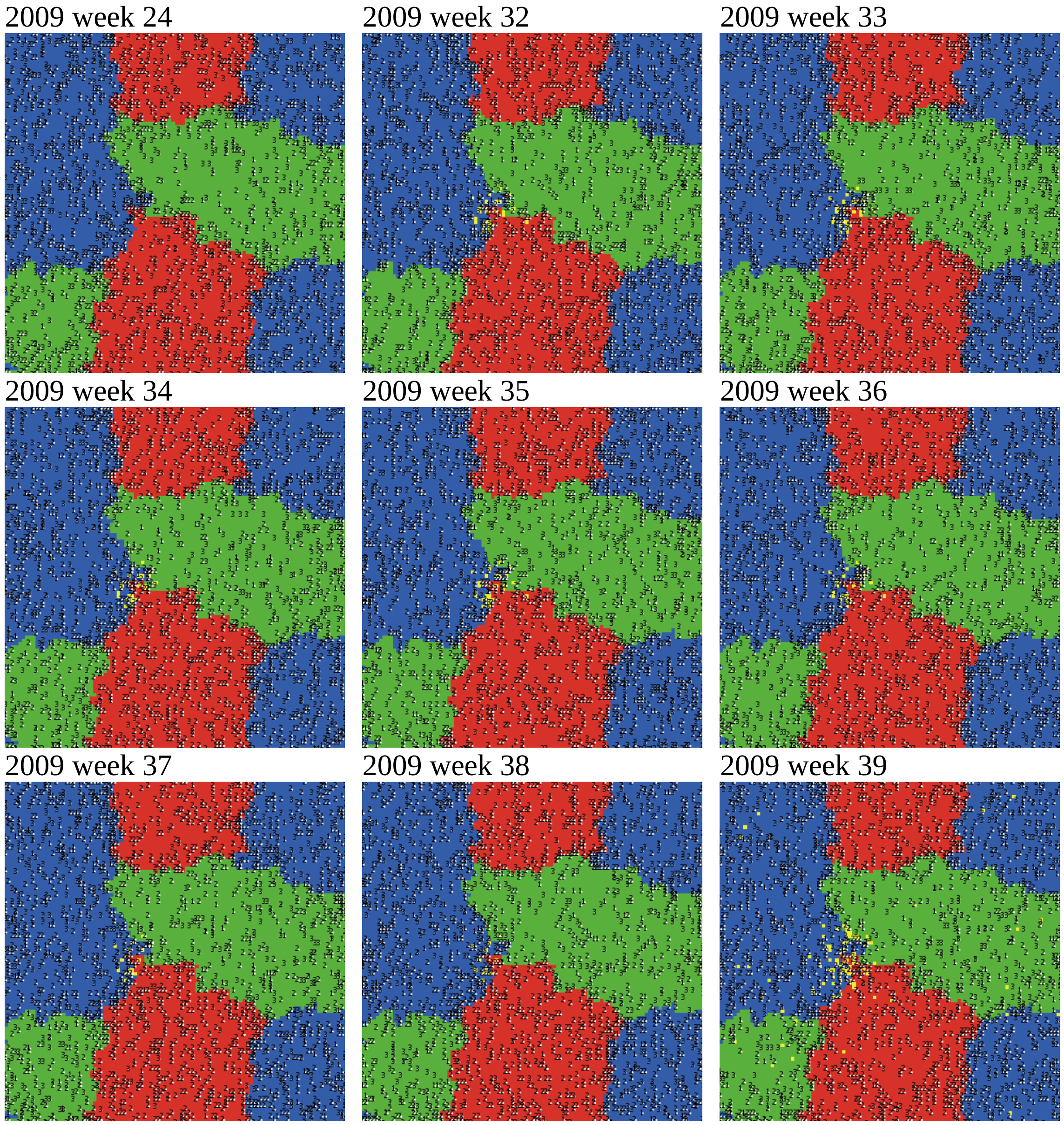

Figure S14 Time series of maps illustrating spatial spread of a tick infestation within the hypothetical 10,000-hectare ranch. One infested head of cattle was introduced at intersection of good (green), fair (red), and poor (blue) tick habitat types during week 25 of 2009. Yellow represents infested landscape cells

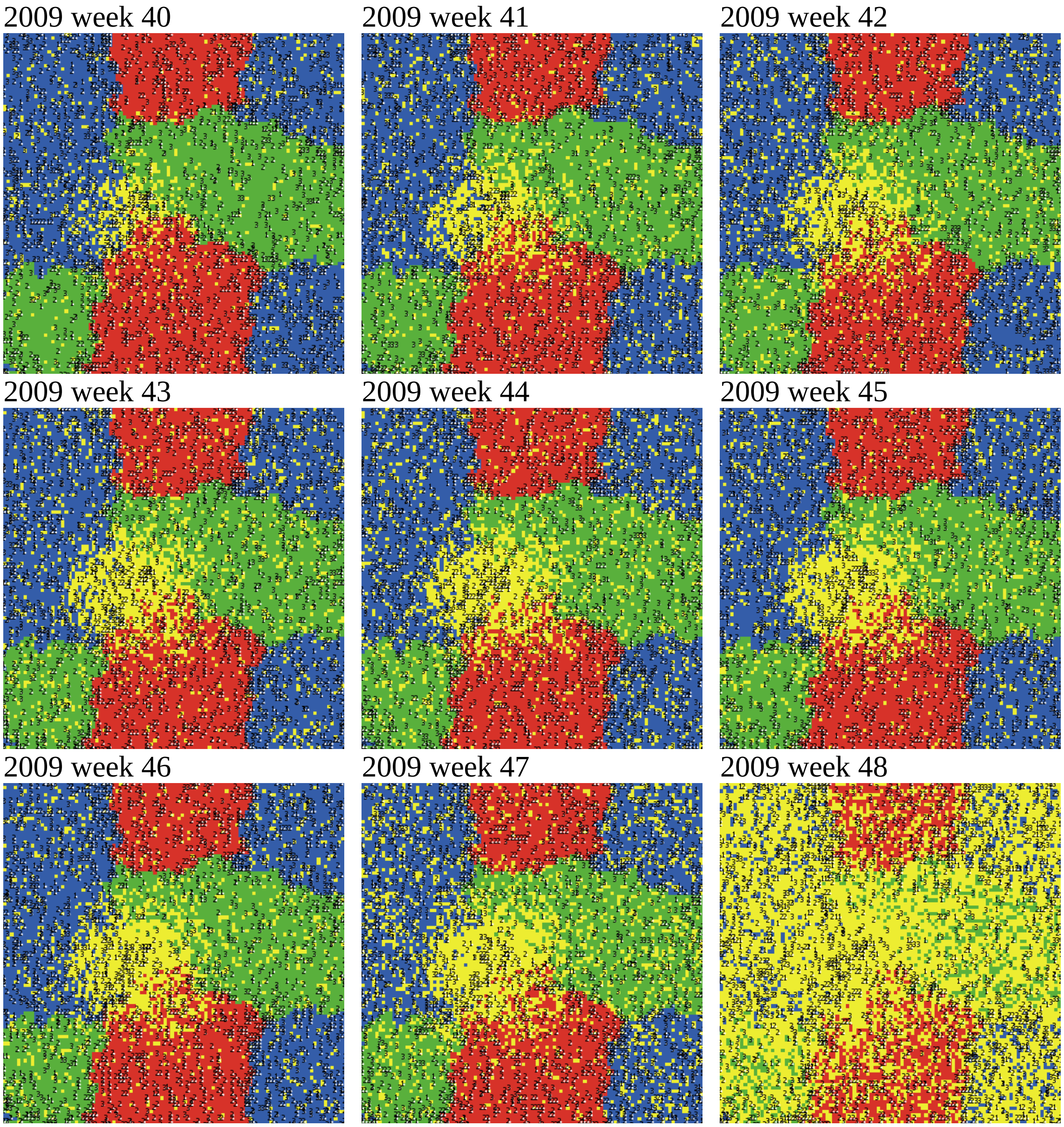

Figure S14 (Cont.) Time series of maps illustrating spatial spread of a tick infestation within the hypothetical 10,000-hectare ranch. One infested head of cattle was introduced at intersection of good (green), fair (red), and poor (blue) tick habitat types during week 25 of 2009. Yellow represents infested landscape cells

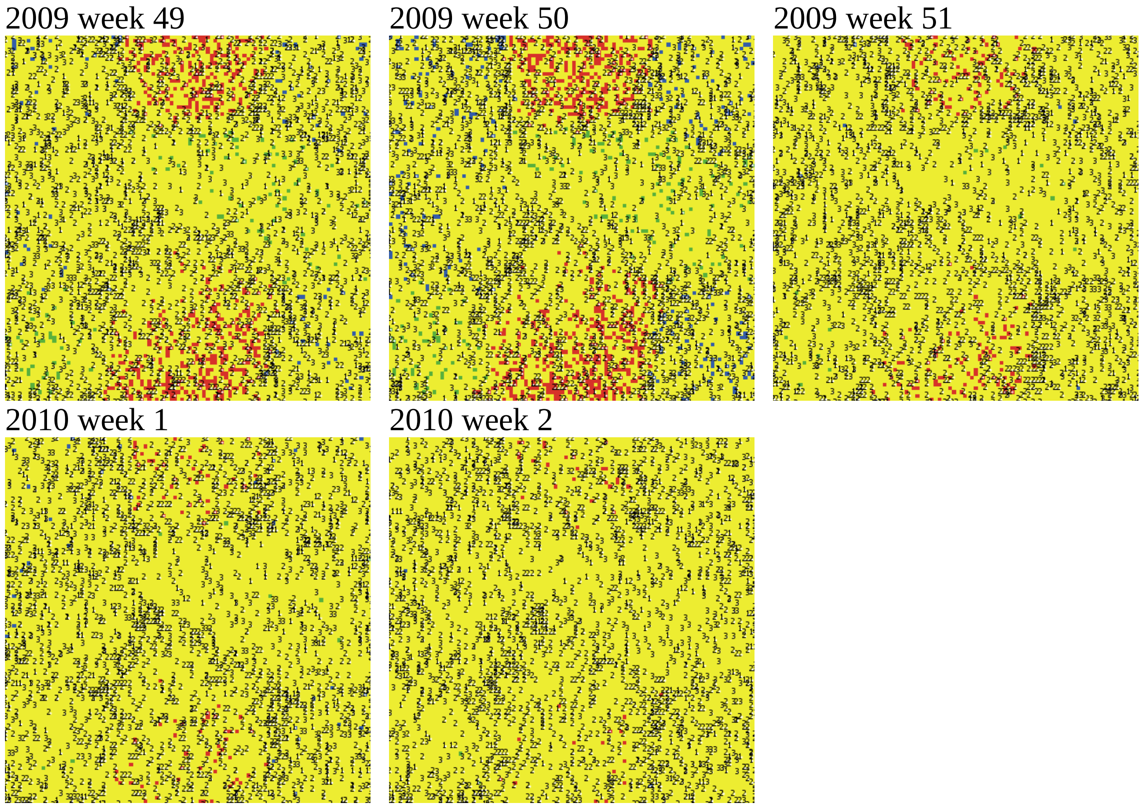

Figure S14 (Cont.) Time series of maps illustrating spatial spread of a tick infestation within the hypothetical 10,000-hectare ranch. One infested head of cattle was introduced at intersection of good (green), fair (red), and poor (blue) tick habitat types during week 25 of 2009. Yellow represents infested landscape cells

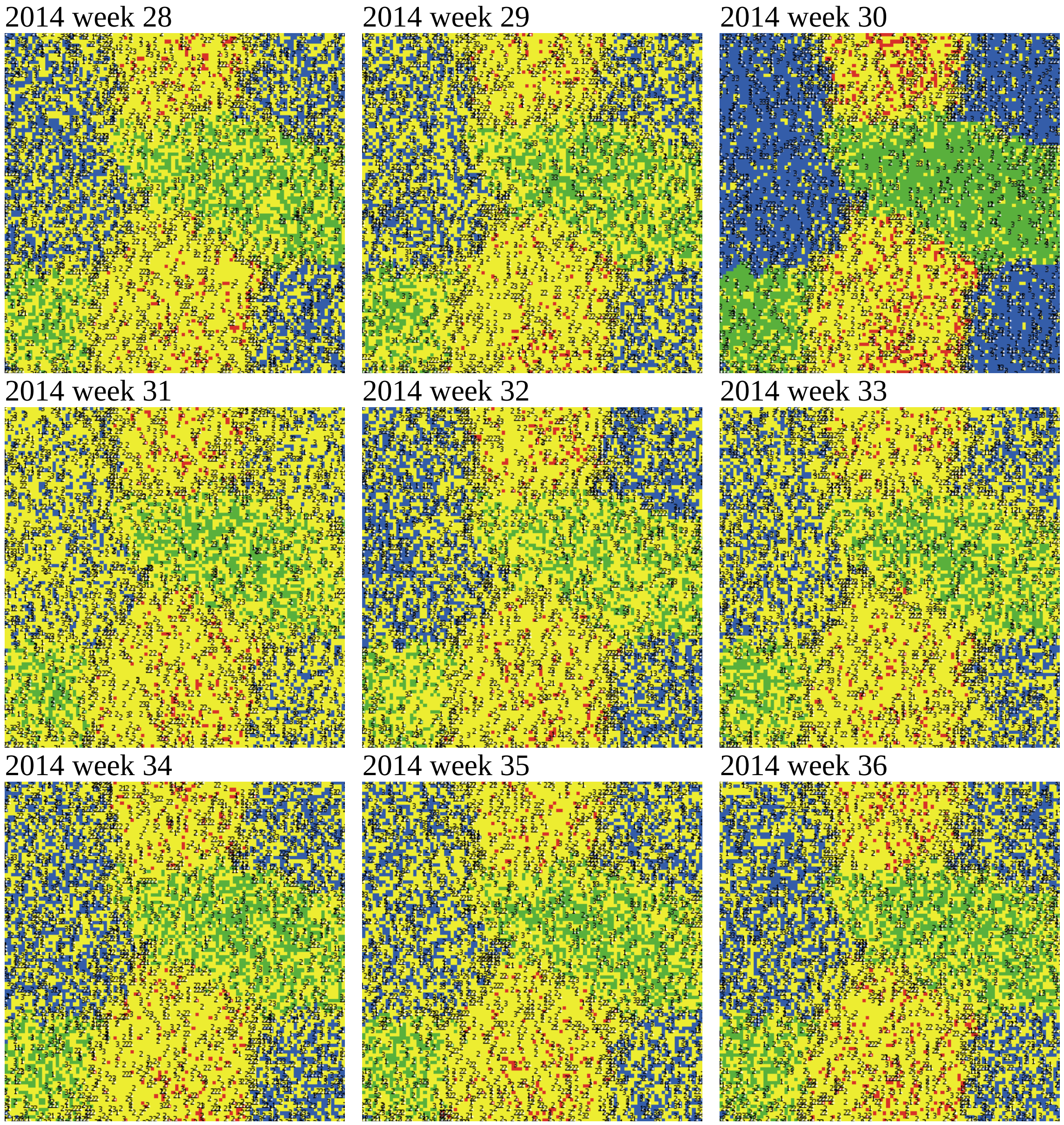

Figure S15 Time series of maps illustrating spatial dynamics of a tick infestation within the hypothetical 10,000-hectare ranch containing good (green), fair (red), and poor (blue) tick habitat types. Acaricide applications capable of complete and continuous elimination of all on-host ticks applied to cattle and nilgai (but not white-tailed deer) were initiated during week 25 of 2009. Yellow represents infested landscape cells

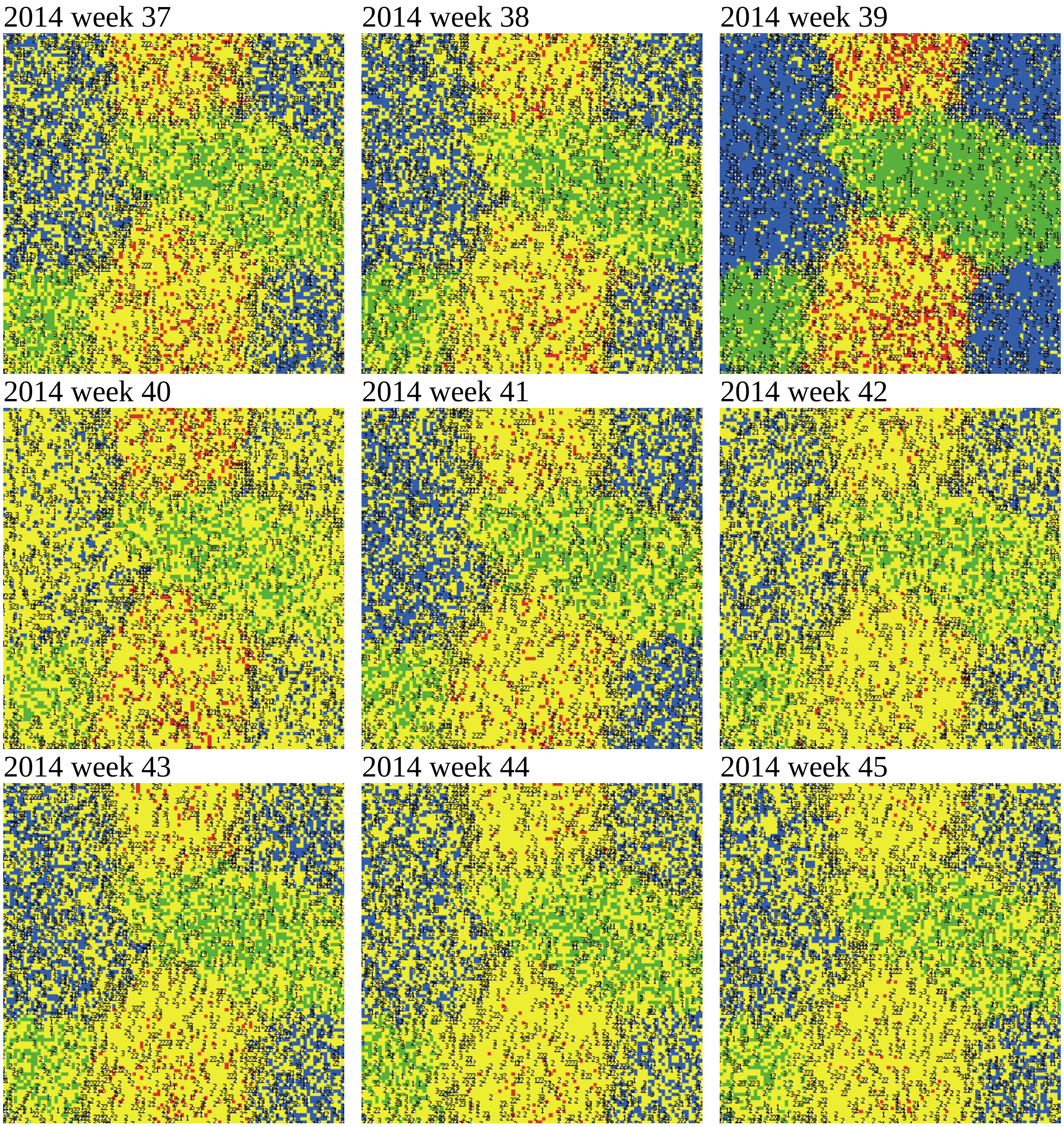

Figure S15 (Cont.) Time series of maps illustrating spatial dynamics of a tick infestation within the hypothetical 10,000-hectare ranch containing good (green), fair (red), and poor (blue) tick habitat types. Acaricide applications capable of complete and continuous elimination of all on-host ticks applied to cattle and nilgai (but not white-tailed deer) were initiated during week 25 of 2009. Yellow represents infested landscape cells

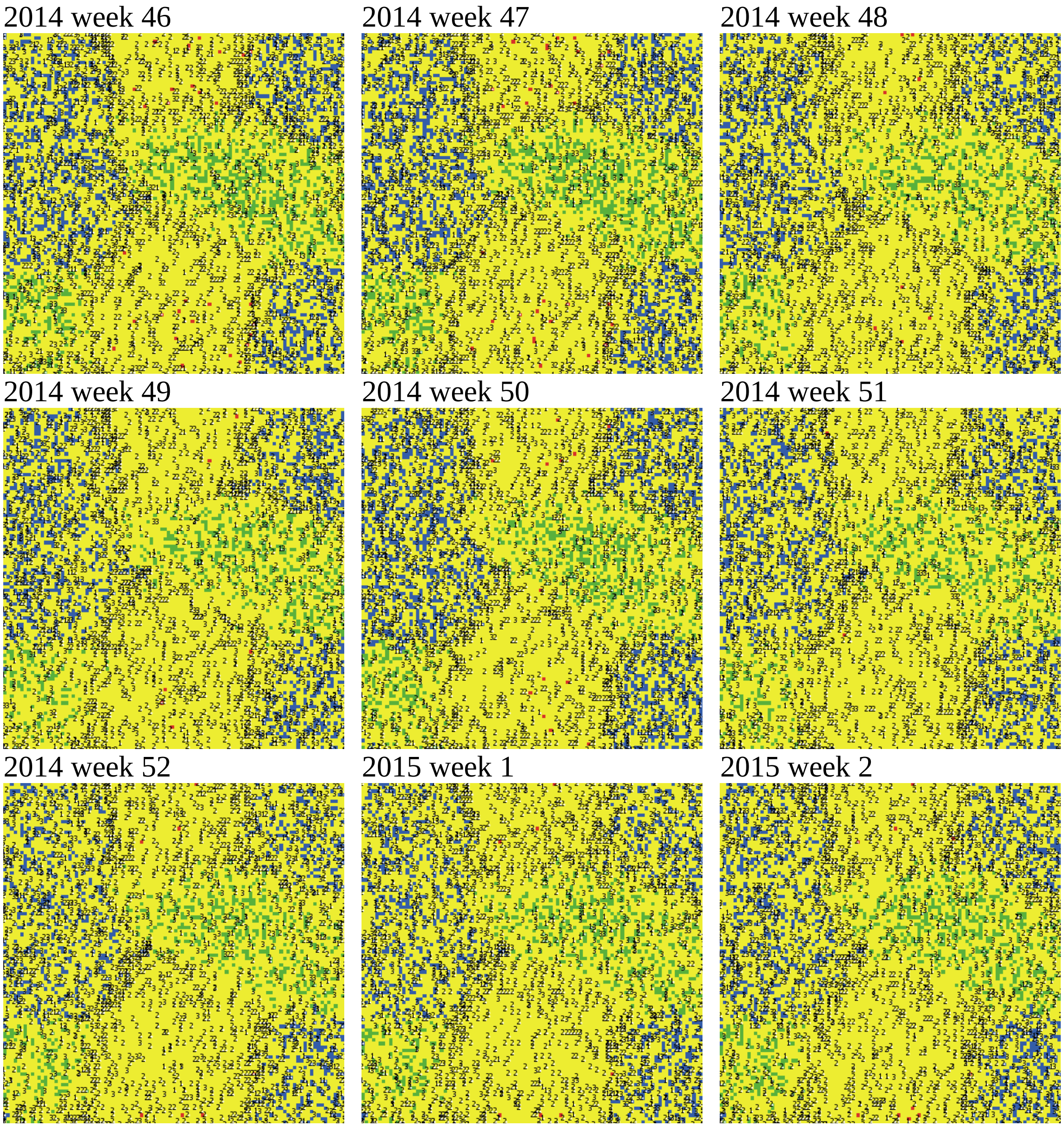

Figure S15 (Cont.) Time series of maps illustrating spatial dynamics of a tick infestation within the hypothetical 10,000-hectare ranch containing good (green), fair (red), and poor (blue) tick habitat types. Acaricide applications capable of complete and continuous elimination of all on-host ticks applied to cattle and nilgai (but not white-tailed deer) were initiated during week 25 of 2009. Yellow represents infested landscape cells

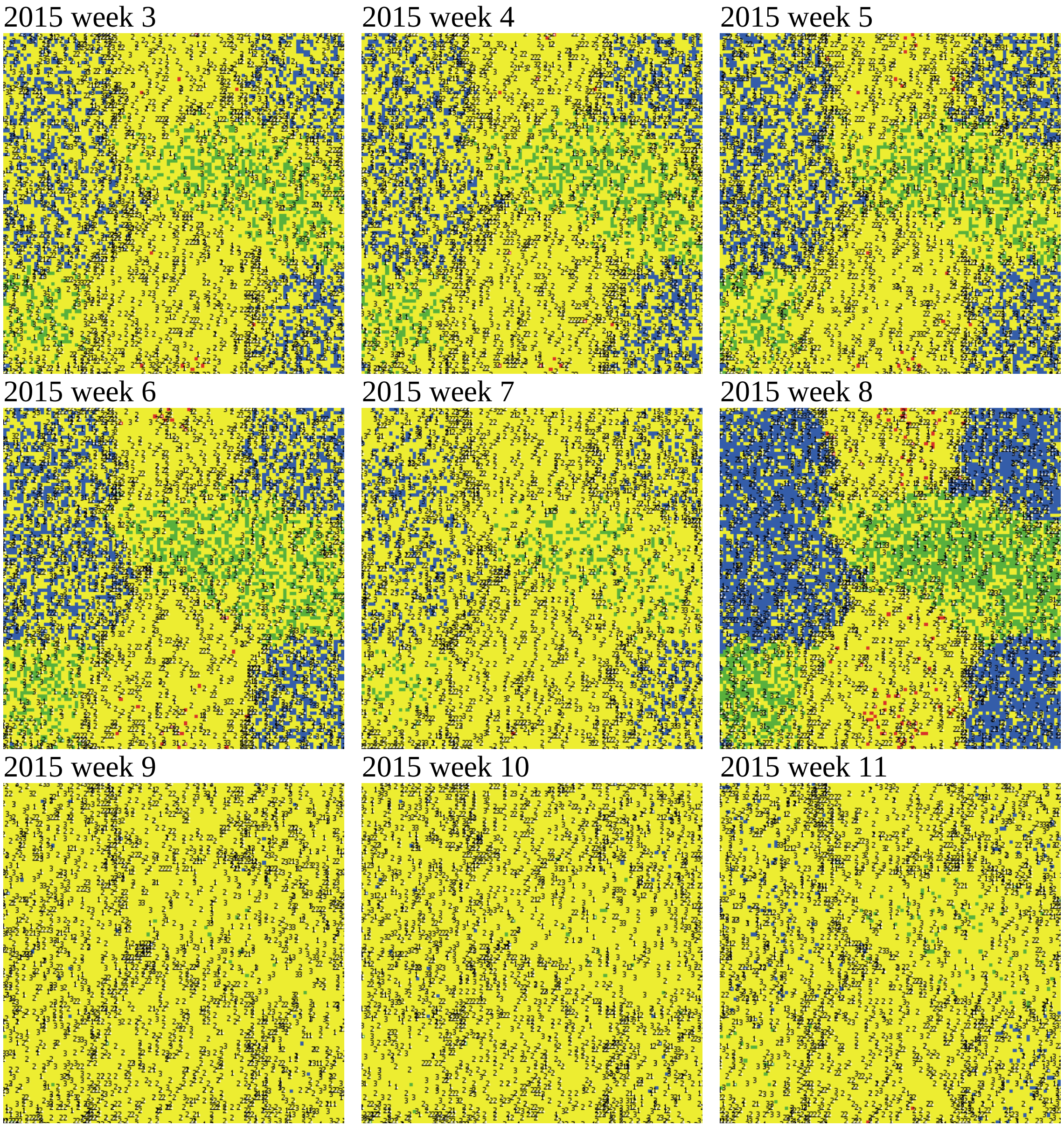

Figure S15 (Cont.) Time series of maps illustrating spatial dynamics of a tick infestation within the hypothetical 10,000-hectare ranch containing good (green), fair (red), and poor (blue) tick habitat types. Acaricide applications capable of complete and continuous elimination of all on-host ticks applied to cattle and nilgai (but not white-tailed deer) were initiated during week 25 of 2009. Yellow represents infested landscape cells

Table S1. List of the parameters used to represent nilgai, cattle, and white-tailed deer as hosts of cattle fever ticks, their baseline values, and their information sources.

Parameters	*Values*	*Reference*
On User Interface		
Cattle/ha	0.0286	[1]
Deer/ha	0.1667	[1]
Nilgai/ha	0.05	[2]
Activity area (ha) cattle	300	[3, 4]
Activity area (ha) deer	675	[3]
Activity area (ha) nilgai	8856	
Relative larvae cattle	1.00	[3, 4]
Relative larvae deer	0.10	[3]
Relative larvae deer	0.50	[3]
Max larvae/host (K)	100	[3]
Hab pref woods* cattle	0.30	[3]
Hab pref mixed brush† cattle	0.10	[3]
Hab pref meadows‡ cattle	0.60	[3]
Hab pref woods* deer	0.20	[3]
Hab pref mixed brush† deer	0.40	[3]
Hab pref meadows‡ deer	0.40	[3]
Hab pref woods* nilgai	0.30	[2]
Hab pref mixed brush† nilgai	0.10	[2]
Hab pref meadows‡ nilgai	0.60	[2]
Pro-areaG (good tick hab)	0.31	The design of this study
Pro-areaF (fair tick hab)	0.28	The design of this study
Pro-areaP (poor tick hab)	0.41	The design of this study
In Setup Code		
Area (ha)	10000	The design of this study
cell-area (ha)	1	[3]
Landscape	hypothetical	The design of this study
versus	real	The design of this study
Year (start year for weather data)	2008	The design of this study
simulation length (years)	11	The design of this study
weather data file	TSDPIWC	NOAA
steps-per-week	30	[3, 4]

*"Woods" refers to Mesquite dominated woody plant community and considered a relatively "Good" climatic environment for CFT to complete the off-host portion of the life cycle and sustain larval survival.

†"Mixed-brush" refers to a community of mixed thorn shrub species and considered a relatively "Fair" climatic environment for CFT to complete the off-host portion of the life cycle and sustain larval survival.

‡"Meadow" refers to uncanopied forage areas and considered a relatively "Poor" climatic environment for CFT to complete the off-host portion of the life cycle and sustain larval survival.

References

1. Cooper SM, Perotto-Baldivieso HL, Owens MK, Meek MG, Figueroa-Pagán M. Distribution and interaction of white-tailed deer and cattle in a semi-arid grazing system. Agric Ecosyst Environ. 2008;127:85–92.
2. Sheffield WJ, Ables ED, Fall BA. Geographic and ecologic distribution of nilgai antelope in Texas. J Wildl Manage. 1971;35:250–7.
3. Wang H-H, Teel PD, Grant WE, Schuster G, Pérez de León AA. Simulated interactions of white-tailed deer (*Odocoileus virginianus*), climate variation and habitat heterogeneity on southern cattle tick (*Rhipicephalus* (*Boophilus*) *microplus*) eradication methods in south Texas, USA. Ecol Model. 2016;342:82-96.
4. Wang H-H, Teel PD, Grant WE, Soltero F, Urdaz J, Ramírez JEP, et al. Simulation tools for assessment of tick suppression treatments of *Rhipicephalus* (*Boophilus*) *microplus* on non-lactating dairy cattle in Puerto Rico. Parasit Vectors. 2019;12:185.
